# Supplementary material for: An oomycete plant pathogen reprograms host pre-mRNA splicing to subvert immunity
Source: Nat Commun. 2017 Dec 12;8:2051. doi: 10.1038/s41467-017-02233-5 (PMC5727057; doi:10.1038/s41467-017-02233-5)
Supplement: Supplementary file 1 — Supplementary Information [file 41467_2017_2233_MOESM1_ESM.pdf]

Supplementary Fig. 1

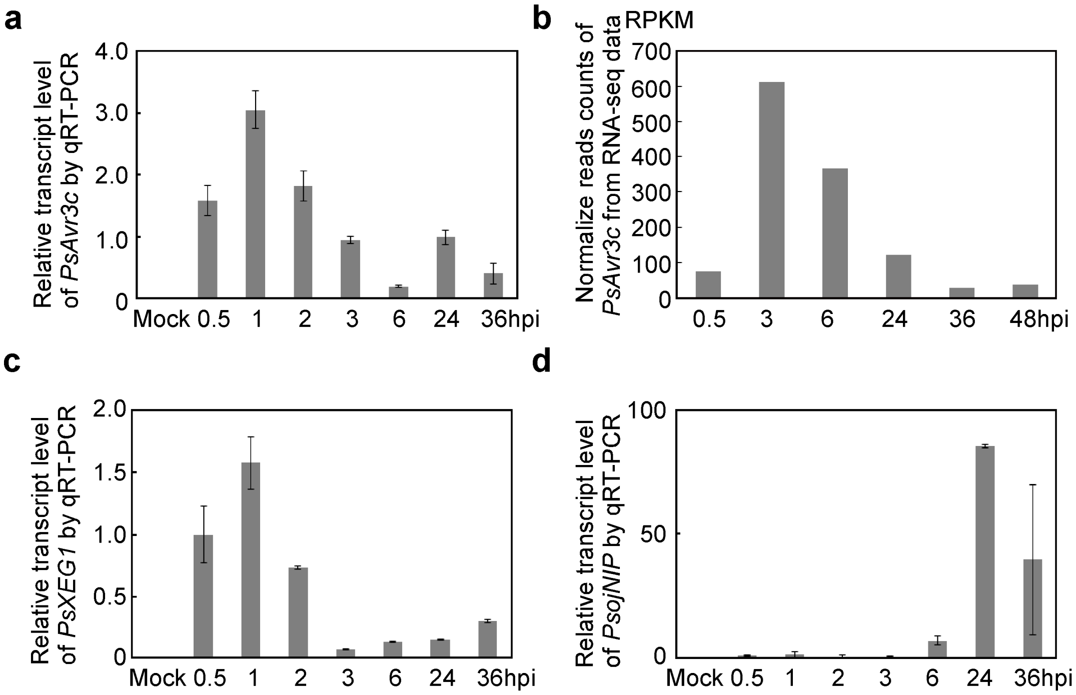

Supplementary Fig. 1. Transcript levels of *PsAvr3c*, *PsXEG1* and *PsojNIP*.

(a) Expression of *PsAvr3c* during *P. sojae* growth and infection of soybean. Total RNA was extracted from susceptible soybean (williams, *rps3c*) root tissues infected with zoospores of *P. sojae* P6497 at 0.5, 1, 2, 3, 6, 24, and 36 hours post-inoculation (hpi). Mock infected samples of root tissues were sampled as a control. Means and standard errors from three replicates are shown. Experiments were repeated three times with similar results.

(b) Transcript level of *PsAvr3c* from RNA-seq data[1]. Total RNA was extracted from *P. sojae* P6497 zoospores infected soybean (williams) roots at 0.5, 3, 6, 24, 36, 48 hpi. The values represent reads per kilo base per million (RPKM) after normalization.

(c) and (d) Transcript levels of control genes *PsXEG1* and *PsojNIP* during *P. sojae* infection. Total RNA was extracted from *P. sojae* P6497 zoospores infected susceptible soybean (williams) roots at 0.5, 1, 2, 3, 6, 24, 36 hpi. Transcript levels were measured by qRT-PCR and normalized. Means and standard errors from three replicates are shown. Experiments were repeated three times with similar results.

Supplementary Fig. 2

a

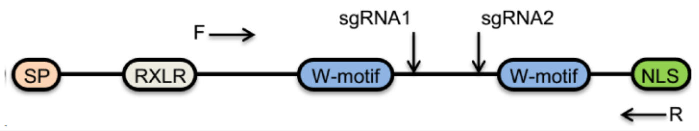

b

Spacer Sequence	Extended Spacer(NNNN[20nt]NGGNNN)	Strand	Score
AGGACGCAAGAAATACGATA	ATGGAGGACGCAAGAAATACGATACGGTGT	sense	0.5635
TCCAGGAACTCTCATCTACA	TGCTTCCAGGAACTCTCATCTACAGGGCAG	sense	0.5451

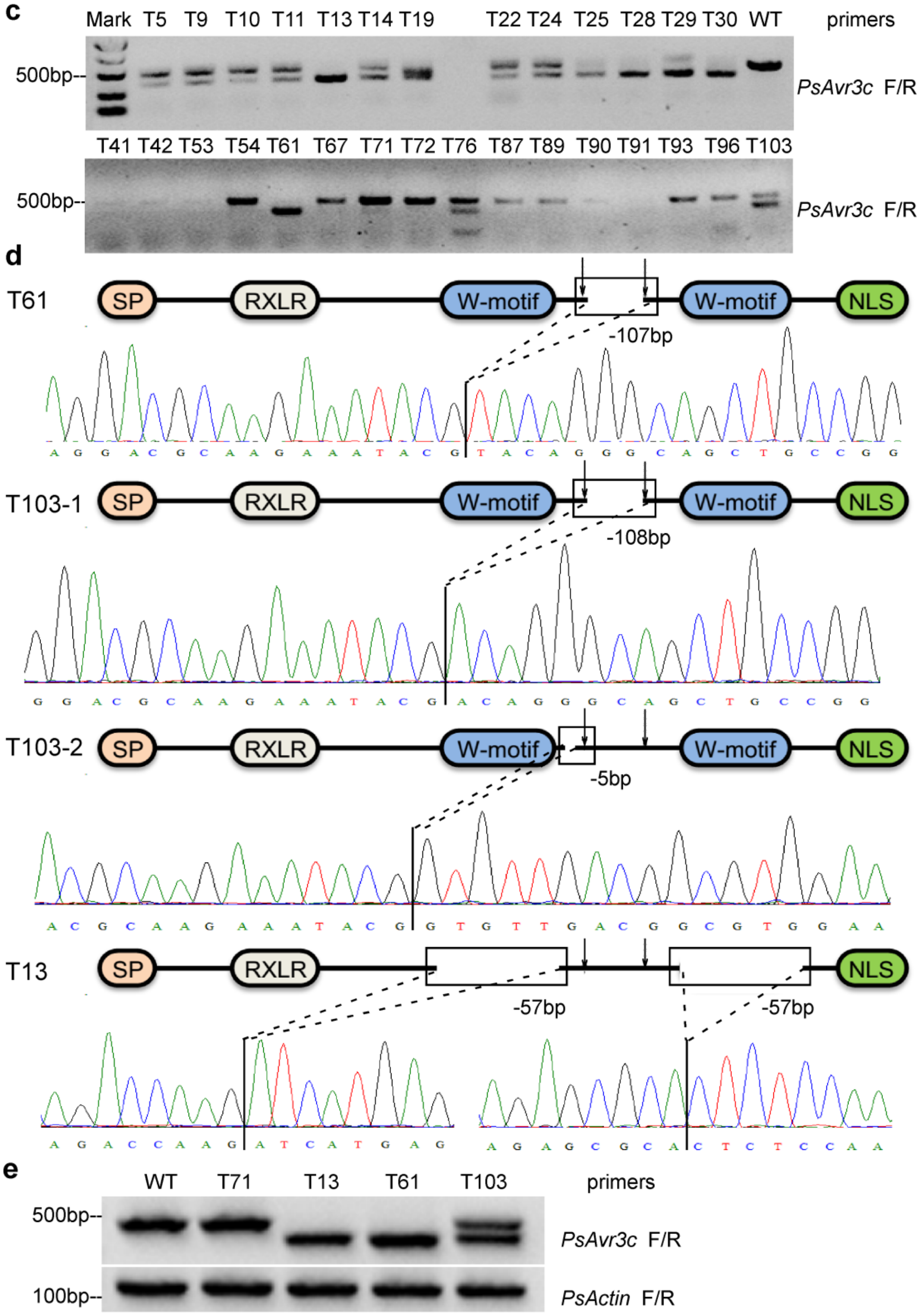

Supplementary Fig. 2. Knock-out of the *PsAvr3c* gene in *P. sojae* using CRISPR/Cas9.

(a) Schematic presentation of avirulence effector PsAvr3c. The positions of the forward (F) and reverse (R) primers used in PCR, and the targeting sites of single guide RNAs (sgRNAs) are indicated with arrows.

(b) Design of two sgRNAs for targeting to *PsAvr3c*. The sgRNA target sites were selected according to the web tool sgRNA Designer (http://www.broadinstitute.org/rnai/public/analysis-tools/sgrna-design). The potential off-target sites were examined using the FungiDB (www.fungidb.org) alignment search tool (BLASTN) against the *P. sojae* genome and visual inspection of the results.

(c) Screening *PsAvr3c* Knockout mutants by PCR amplification of genomic DNA. Agarose gel shows PCR bands amplified across sgRNA1 and sgRNA2 using genomic DNA extracted from different transformants, demonstrating that transformants appeared as homozygous and heterozygous for mutations to *PsAvr3c*, while others remained wild type.

(d) Sanger sequencing traces confirm that *PsAvr3c* gene is disrupted in CRISPR/Cas9 mutated *P. sojae* strains. The sites of sgRNAs are indicated by downward arrows. Mutants T13 and T61 are homozygous. A 107 bp fragment is missing between two sgRNA sites in T61, compared to wild-type. For T13, two independent 57 bp deletion events occur in each of the sgRNA sites. T103 is a heterozygote, with an 108 bp and an 5 bp deletion in each of the two alleles of *PsAvr3c.*

(e) Verify *PsAvr3c* Knockout mutants by PCR amplification of cDNA. *P. sojae* actin gene (VMD GeneID: 108986) was used as an internal reference. Total RNA was extracted from *P. sojae* mutants mycellia. The agarose gel shows a PCR result of *PsAvr3c* loci from individual *P. sojae* mutants. Mutants T13 and T61 are homozygous, whereas T103 is a heterozygote. WT represents wild type *P. sojae* strain P6497. The line T71 is a transformant without *PsAvr3c* editing events.

Supplementary Fig. 3

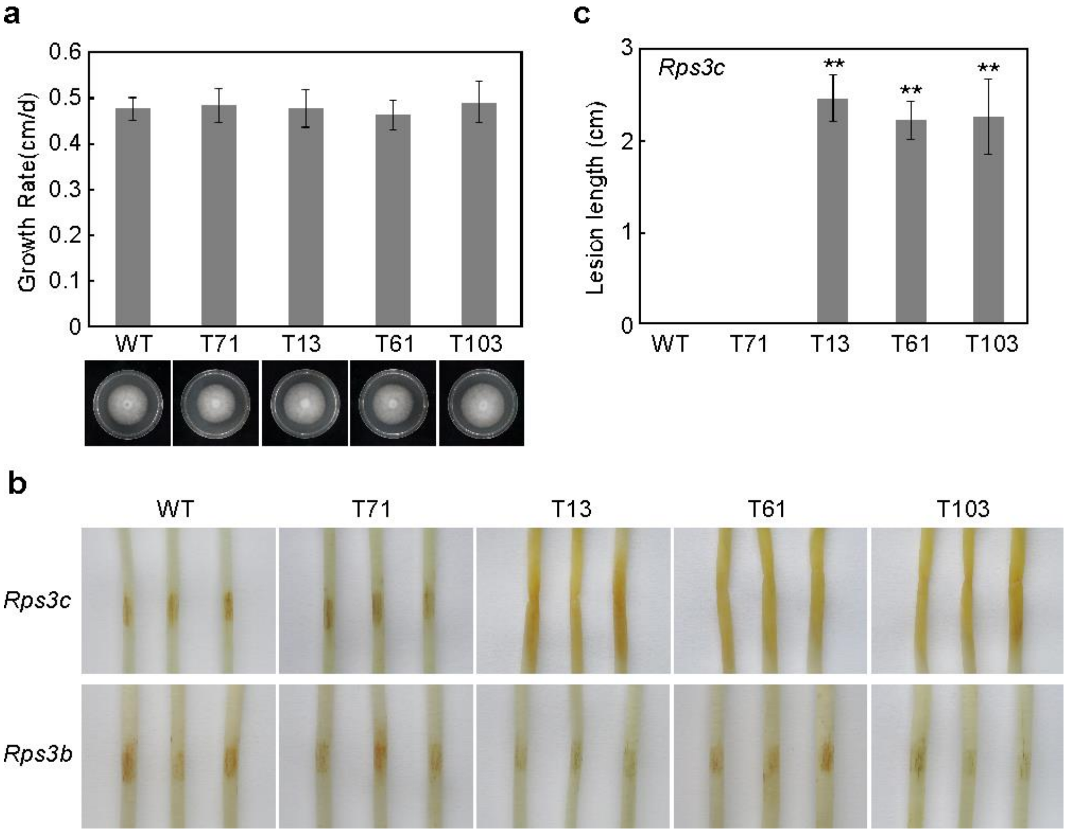

Supplementary Fig. 3. The *PsAvr3c* Knock-out mutation does not affect *Phytophthora sojae* growth *in vitro* and verify *PsAvr3c* Knockout mutants by phenotype.

(a) Growth of *PsAvr3c* Knock-out mutants. Wild-type (WT), control (T71) and mutant transformants were incubated on 10% vegetable juice (V8) medium at 25°C for 4 days and then photographs were taken. Colony growth rate was calculated based on the diameters of colonies. Means and standard errors from three replicates are shown. The experiment was replicated three times with similar results.

(b) Symptoms of etiolated soybean seedlings infected by *P. sojae* strains. Soybean lines that carrying single resistance genes *Rps3c* or *Rps3b* were inoculated with wild-type and *PsAvr3c* mutant strains of *P. sojae*. T13, T61 and T103 are three knock-out mutants and T71 is a transformant without *PsAvr3c* editing events. Photographs were taken at two days post-inoculation (dpi). Experiments were repeated three times with similar results.

(c) Measurements of lesion lengths on resistant soybean (*Rps3c*). Means and standard errors of lesion lengths from three replicates are shown (** $P<0.01$; $n=3$, one-way ANOVA).

Supplementary Fig. 4

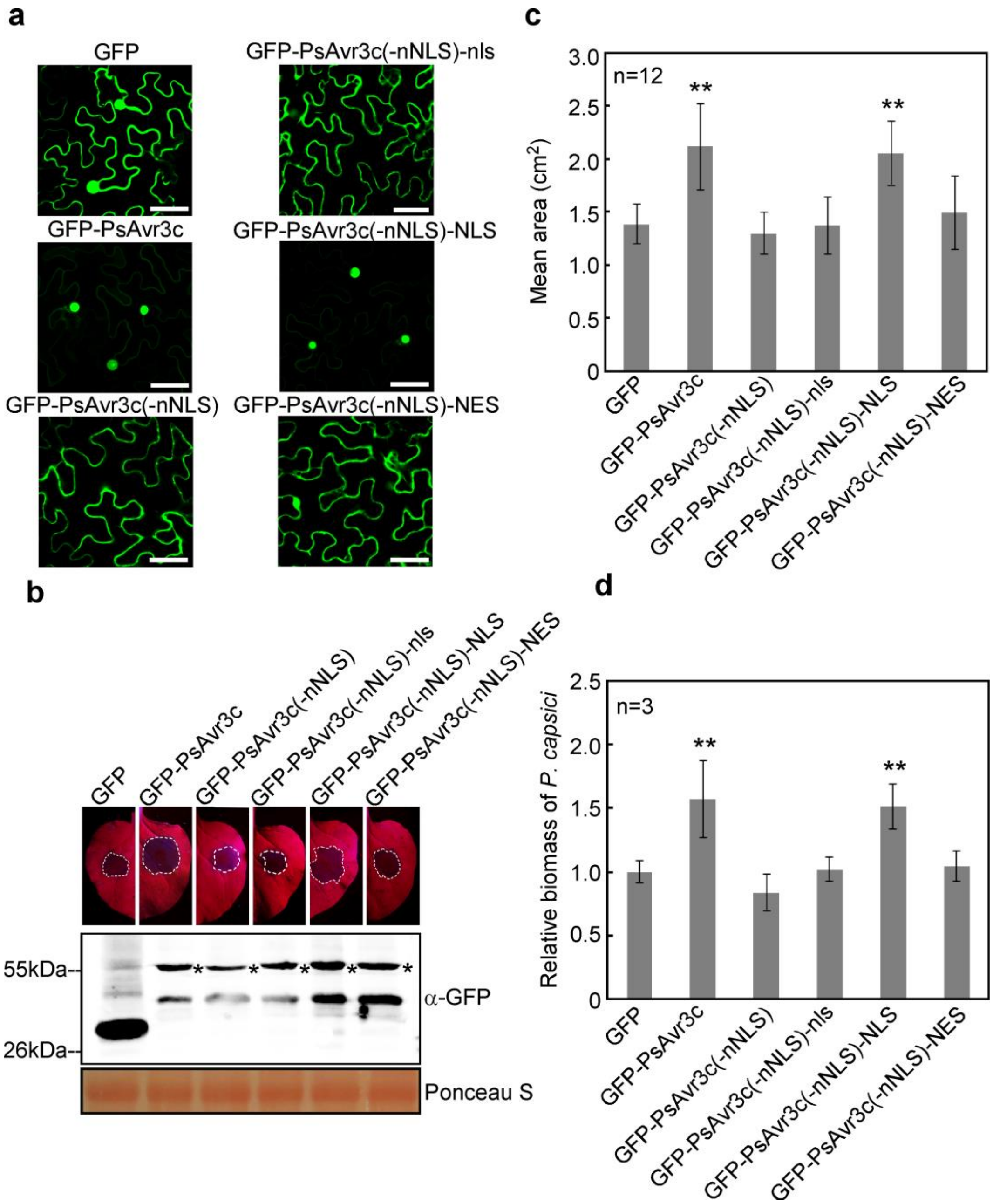

Supplementary Fig. 4. PsAvr3c possesses a functional NLS, which is required for full virulence.

(a) PsAvr3c nuclear localization signal (NLS) is functional. Shown are confocal images of *N. benthamiana* epidermal cells transiently expressing the GFP, GFP-PsAvr3c, GFP-PsAvr3c(-nNLS) (deleted native NLS), GFP-PsAvr3c(-nNLS)-nls (replace native NLS with nonfunctional NLS), GFP-PsAvr3c(-nNLS)-NLS (replace native NLS with functional NLS), GFP-PsAvr3c (-nNLS)-NES (replace native NLS with functional NES). Photographs were taken at 48 hpi. Scale bar represents 50 μm.

(b) Nuclear localization is important for PsAvr3c induced susceptibility. The *N. benthamiana* leaves were infiltrated to express the indicated constructs, and 36 hours later inoculated with *P. capsici*. Photographs were taken at 30 hpi under UV light. Dashed lines indicate the lesion areas. Protein expression was confirmed by western blotting using anti-GFP antibody. Protein bands corresponding to the PsAvr3c constructs are indicated by asterisks, protein loading is visualized by Ponceau stain.

(c) Average lesion sizes of infected *N. benthamiana* leaves. Lesion sizes (cm^2) were measured at 30 hpi. Means and standard errors from separate measurements are shown (** $P<0.01$; one-way ANOVA). Means and standard errors from twelve replicates are shown. Experiments were repeated three times with similar results.

(d) Relative biomass quantification of inoculated *N. benthamiana* leaves. DNA from *P. capsici* infected regions was isolated at 30 hpi and primers specific for the *N. benthamiana* and *P. capsici* actin gene were used for qRT-PCR. qRT-PCR was performed and normalized to GFP (** $P<0.01$; one-way ANOVA). Means and standard errors from three replicates are shown. Three independent experiments gave similar results.

Supplementary Fig. 5

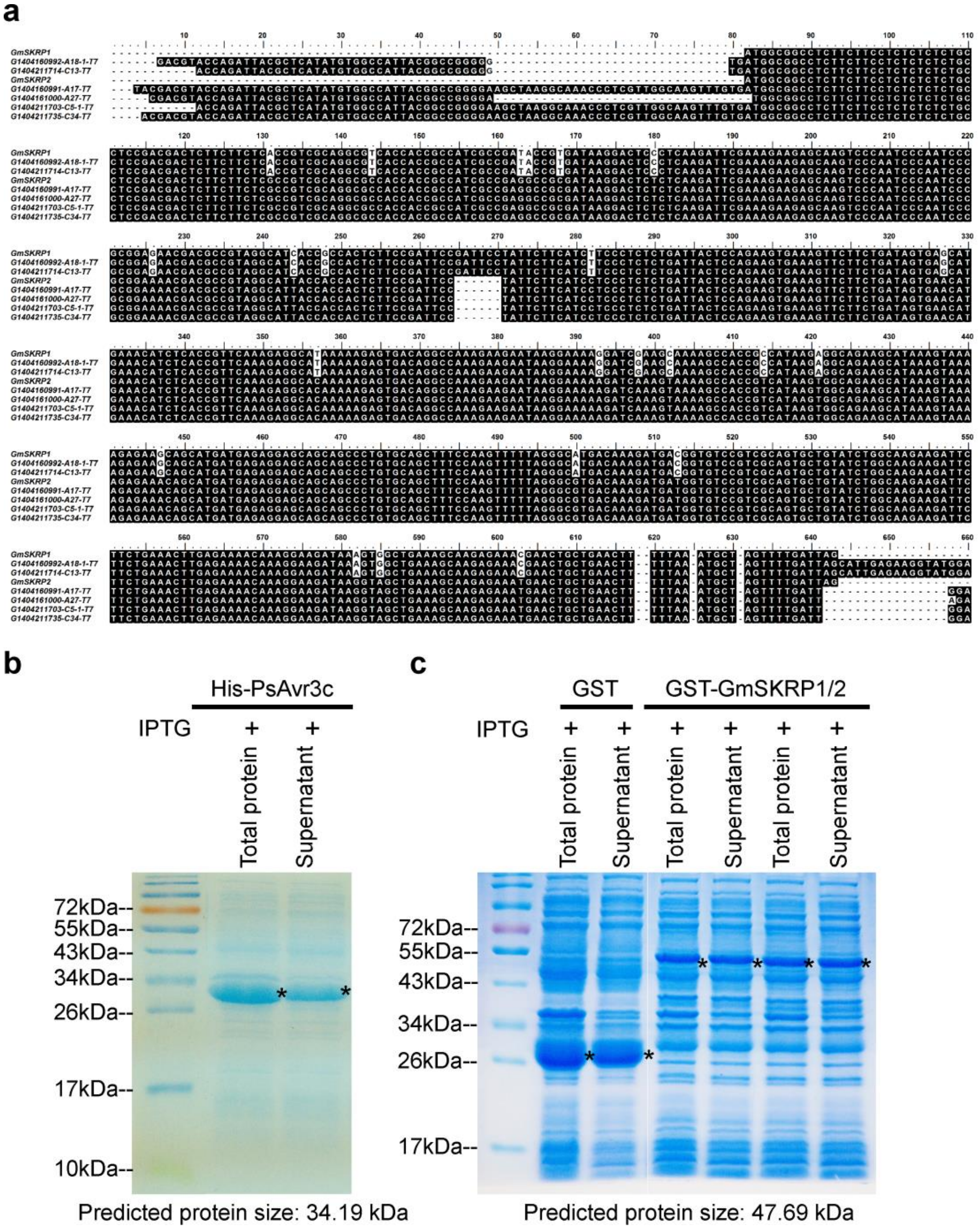

Supplementary Fig. 5. GmSKRP1/2 sequences from Y2H and recombinant proteins expression in *E. coli*.

(a) The raw sequences of different prey plasmids aligned to predicted *GmSKRP1/2* coding sequence. BioEdit was used for the multiple alignment of the sequence.

(b) Coomassie Brilliant Blue staining of an SDS-PAGE gel. Left lane: molecular weight markers; right lanes: total protein and supernatant samples from His-PsAvr3c. The recombinant PsAvr3c protein band is indicated by asterisk.

(c) Shown is an SDS-PAGE analysis of GST and GST-GmSKRP1/2 proteins expression, with the gel stained with Coomassie Brilliant Blue. Left lane: molecular weight markers; right lanes: total protein and supernatant samples from GST and GST-GmSKRP1/2. The recombinant protein bands are indicated by asterisks.

Supplementary Fig. 6

a

PsAvh52 full protein sequence

MRLTSILVLVIAATFHHTTGTALTLTKDSKAGIANGDSPASGDFIDANSARLLRRVEKDKVDYE
QDEQRSFGALKDAVKKLNPVTAVKKFFKQRAKRKKVIQTARDADNNLAWAMKEVYKAAN

NLS

b

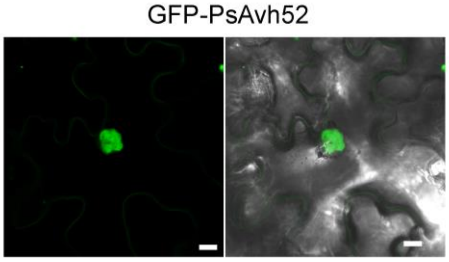

c

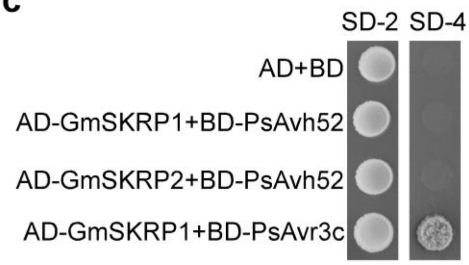

Supplementary Fig. 6. *P. sojae* effector PsAvh52 can not interact with GmSKRPs proteins.

(a) The full predicted amino acid sequence of PsAvh52. Nuclear localization signal is highlighted in blue.

(b) The localization pattern of GFP-PsAvh52. Confocal imaging shows that GFP-PsAvh52 localizes in the cell nuclei. Scale bar represents 10 μm.

(c) PsAvh52 cannot interact with GmSKRPs in yeast. The BD-PsAvh52 and BD-PsAvr3c (as positive control) were co-transformed with AD-GmSKRPs into AH109, respectively. Yeast transformants were grown on SD/-Trp/-Leu (SD-2) and selected on SD/-Trp/-Leu/-His/-Ade (SD-4), the plates were photographed 5 days after inoculation.

Supplementary Fig. 7

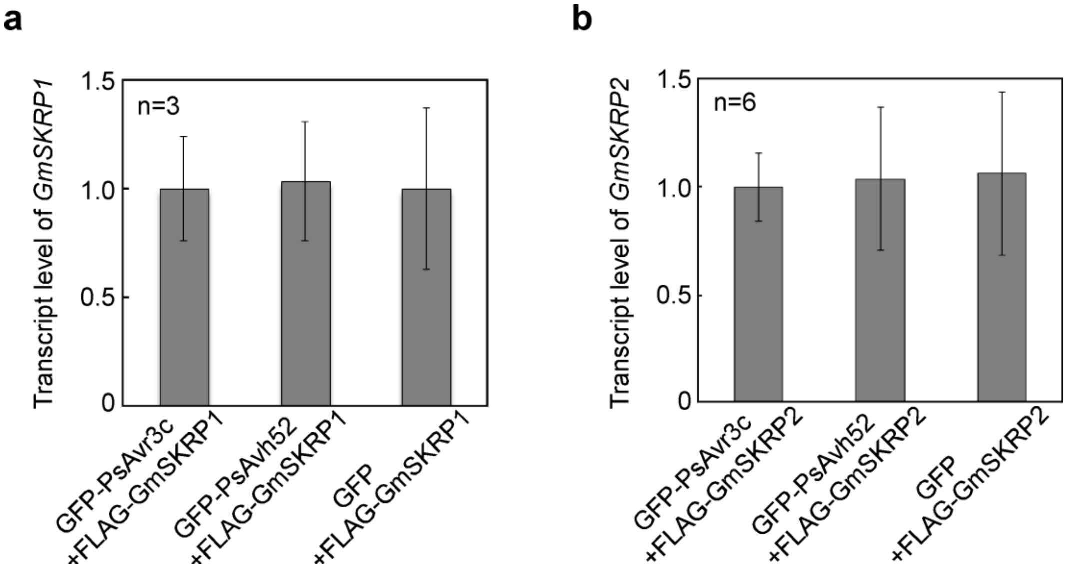

Supplementary Fig. 7. The ectopic transcript level of FLAG-GmSKRP1/2 is not affected by co-expression with GFP-PsAvr3c, GFP-PsAvh52 and GFP in *N. benthamiana.*

Total RNA from samples shown in Fig. 3a was extracted at 48 hpi. Analysis by qRT-PCR was performed using specific primers to measure transcript level of GmSKRP1 (a) and GmSKRP2 (b). The *N. benthamiana* actin gene was used as internal control gene. Means and standard errors from three replicates (a) and six replicates (b) are shown. Experiments were repeated three times with similar results.

Supplementary Fig. 8

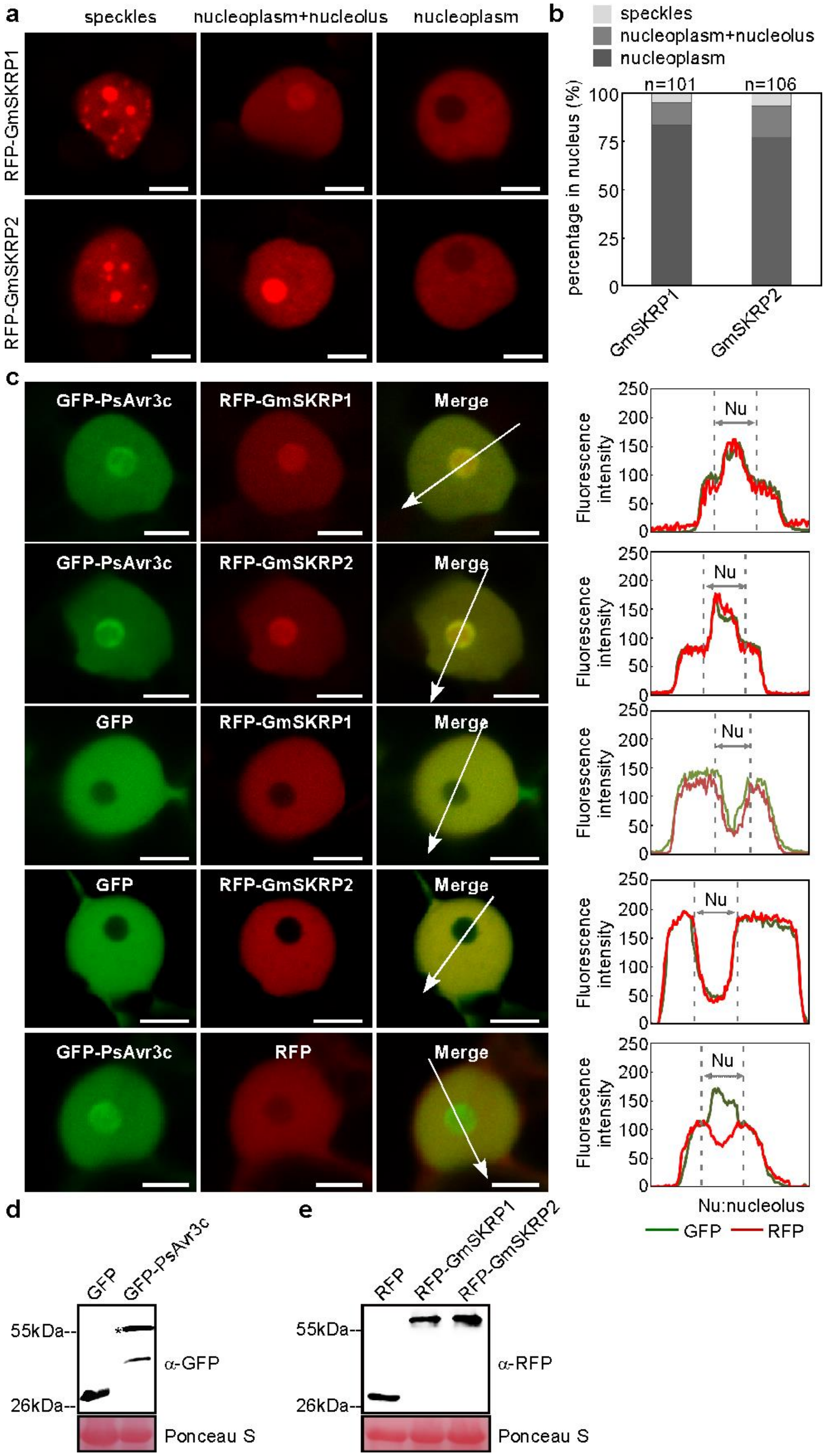

Supplementary Fig. 8. PsAvr3c relocates GmSKRP1/2 from nucleoplasm to nucleolus.

(a) The localization pattern of RFP-GmSKRP1/2. Confocal imaging shows that RFP-GmSKRP1/2 proteins are diffusely distributed in the speckles, nucleoplasm and nucleolus, nucleolus. Scale bar represents 5 μm.

(b) Statistic analysis of (a). RFP-GmSKRP1/2 fusion proteins predominantly accumulated in the nucleoplasm, with a low proportion of nuclear speckles or nucleoplasm and nucleolus localization.

(c) GmSKRPs are relocated from nucleoplasm to nucleolus in the presence of PsAvr3c. Confocal images of *N. benthamiana* leaf epidermal cell nuclei transiently expressing the RFP-GmSKRP1/2 with GFP-PsAvr3c demonstrate that GmSKRPs proteins were relocated from nucleoplasm to nucleolus when they are co-expressed with GFP-PsAvr3c but not with GFP control. In contrast, RFP protein localization does not change when it is co-expressed with GFP-PsAvr3c. Scale bar represents 5 μm.

(d-e) Immunoblot analyses of GFP, GFP-PsAvr3c, RFP, RFP-GmSKRPs proteins. Total proteins were extracted at 48 hpi. Protein expression was confirmed by immuno-blotting using an anti-GFP or anti-RFP antibody. Protein bands are indicated by asterisks, protein loading is indicated by Ponceau stain.

Supplementary Fig. 9

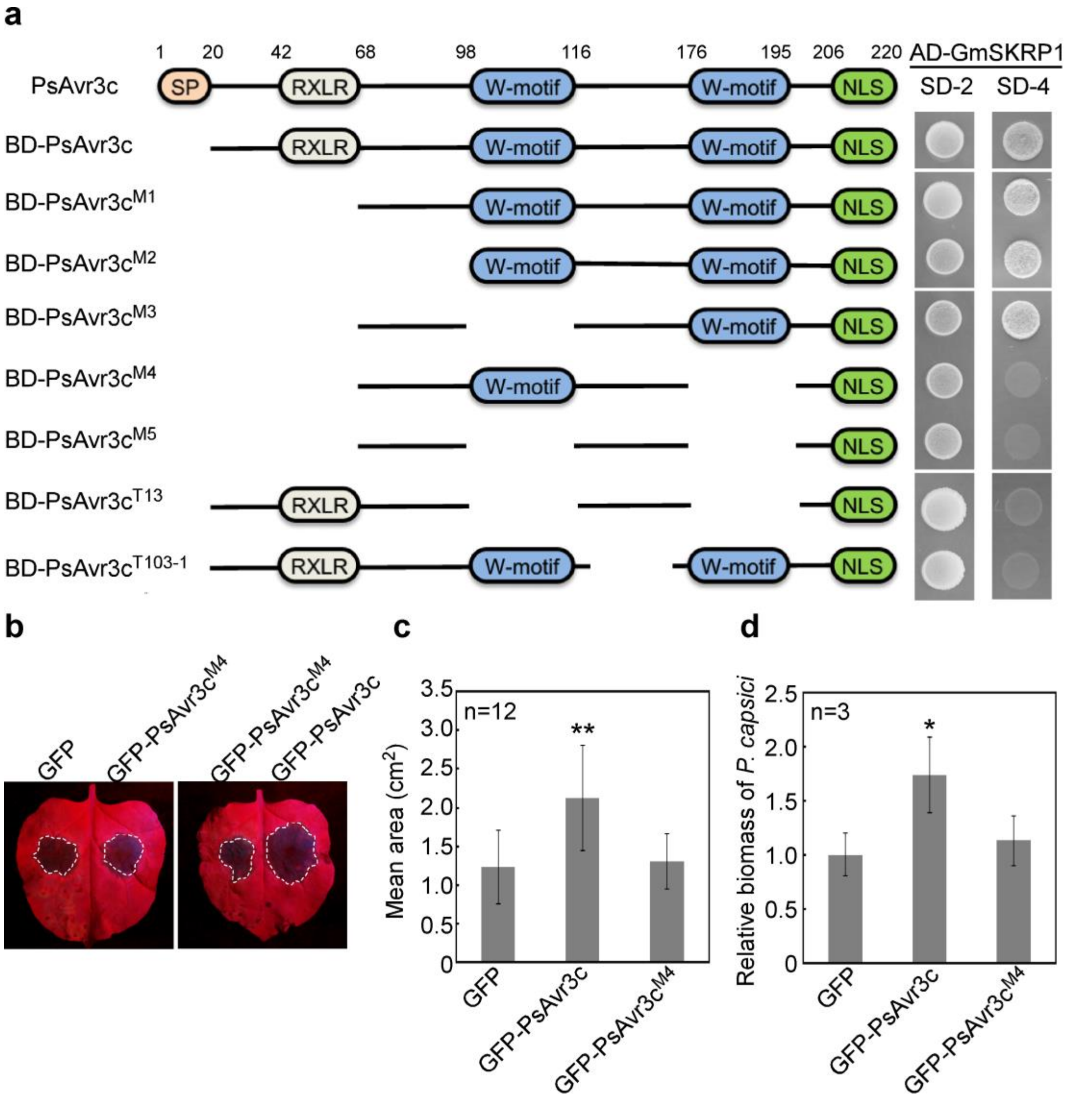

Supplementary Fig. 9. Screening PsAvr3c mutants and testing the interaction between PsAvr3c mutants and GmSKRP1 in yeast.

(a) Verifying the interaction between PsAvr3c mutants and GmSKRP1 in yeast. Schematic drawings of PsAvr3c and mutants are shown on the left. The interaction between PsAvr3c mutants and GmSKRP1 are shown on the right. PsAvr3c mutants and GmSKRP1 were cloned into bait plasmid pGBKT7 (BD) and prey plasmid pGADT7 (AD) vectors plasmid, respectively. Yeast transformants were grown on SD/-Trp/-Leu (SD-2) and selected on SD/-Trp/-Leu/-His/-Ade (SD-4), the plates were photographed at 5 days after inoculation. Experiments were repeated three times with similar results.

(b) *P. capsici* infection assay on *N. benthamiana* leaves expressing GFP-PsAvr3c or GFP-PsAvr3c^{M4}. *P. capsici* mycellia were inoculated on the infiltrated leaves at 36 h after Agro-infiltration. The photographs were taken at 30 hpi under UV light. Dashed lines show the lesion areas.

(c) Average lesion size of inoculated leaves. Lesion size (cm^2) were measured at 30 hpi. Means and standard errors from separate measurements are shown (** $P<0.01$; one-way ANOVA). Bars indicate standard errors from twelve replicates. This experiment was repeated three times with similar results.

(d) Relative biomass quantification of inoculated *N. benthamiana* leaves. DNA from *P. capsici* infected regions was isolated at 30 hpi and primers specific for the *N. benthamiana* and *P. capsici* actin gene were used for qRT-PCR. qRT-PCR was performed and normalized to GFP. Means and standard errors from three replicates are shown (* $P<0.05$; one-way ANOVA). Three independent experiments gave similar results.

Supplementary Fig. 10

a

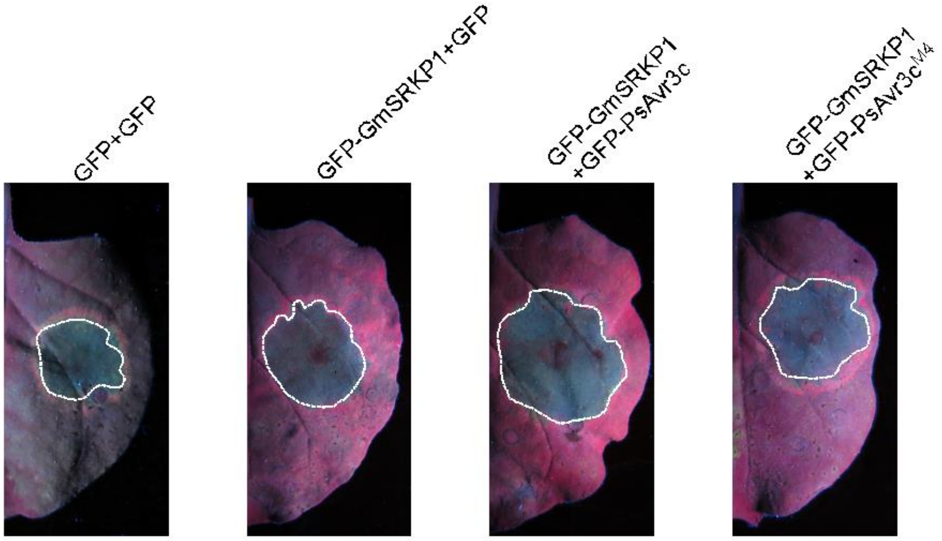

b

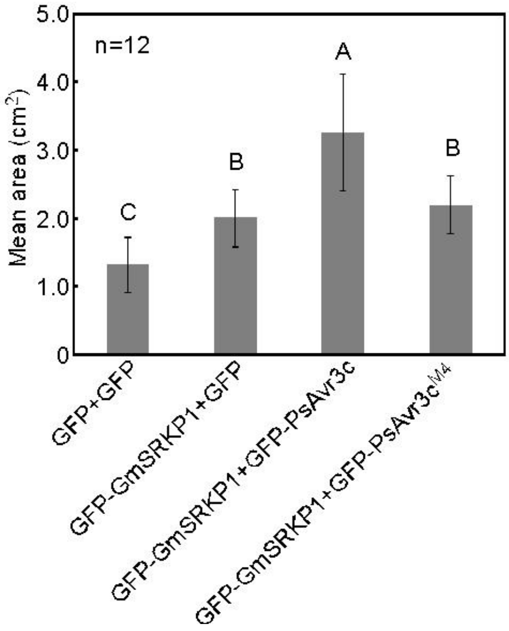

c

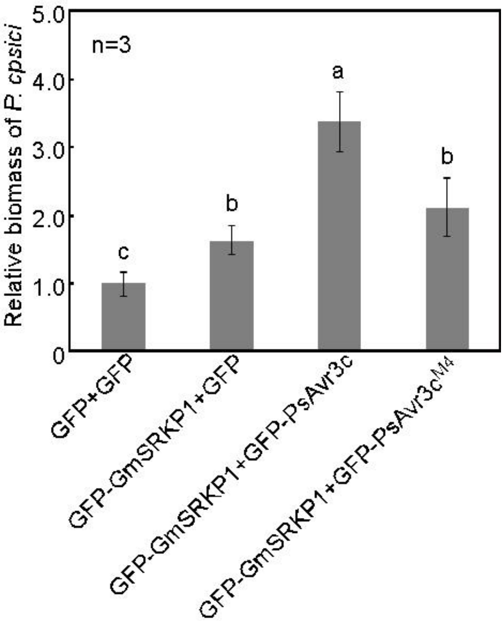

Supplementary Fig. 10. Co-expression of GFP-PsAvr3c and GFP-PsAvr3c^{M4} with GFP-GmSKRP1 in *N. benthamiana.*

(a) Typical infection areas of inoculated *N. benthamiana* leaves. *P. capsici* mycelia were inoculated on leaves at 36 h after infiltration. Co-expression of GFP-PsAvr3c and GFP-GmSKRP1 in leaves results in greater susceptibility to *P. capici* compared to the level of susceptibility induced by expression of the GmSKRP1 or PsAvr3c alone. This effect was not observed when the PsAvr3c^{M4} mutant was used to in place of wild-type PsAvr3c. Pictures were taken at 30 hpi under UV light. Dashed lines indicate the lesion areas. Three independent experiments gave similar results.

(b) Average lesion areas of *N. benthamiana* leaves inoculated by *P. capsici.* Lesion areas (cm^2) were measured at 30 hpi. Means and standard errors from three replicates are shown. Letters represent significant differences as measured (** $P<0.01$; one-way ANOVA). Bars indicate standard errors from twelve replicates. The experiment was replicated three times with similar results.

(c) Relative biomass of inoculated *N. benthamiana* leaves. DNA from *P. capsici* infected leaves was isolated at 30 hpi, and primers specific for the *N. benthamiana* and *P. capsici* actin gene were used for qRT-PCR biomass assay. qRT-PCR was performed and normalized. Means and standard errors from three replicates are shown (* $P<0.05$; one-way ANOVA). Three independent experiments gave similar results.

Supplementary Fig. 11

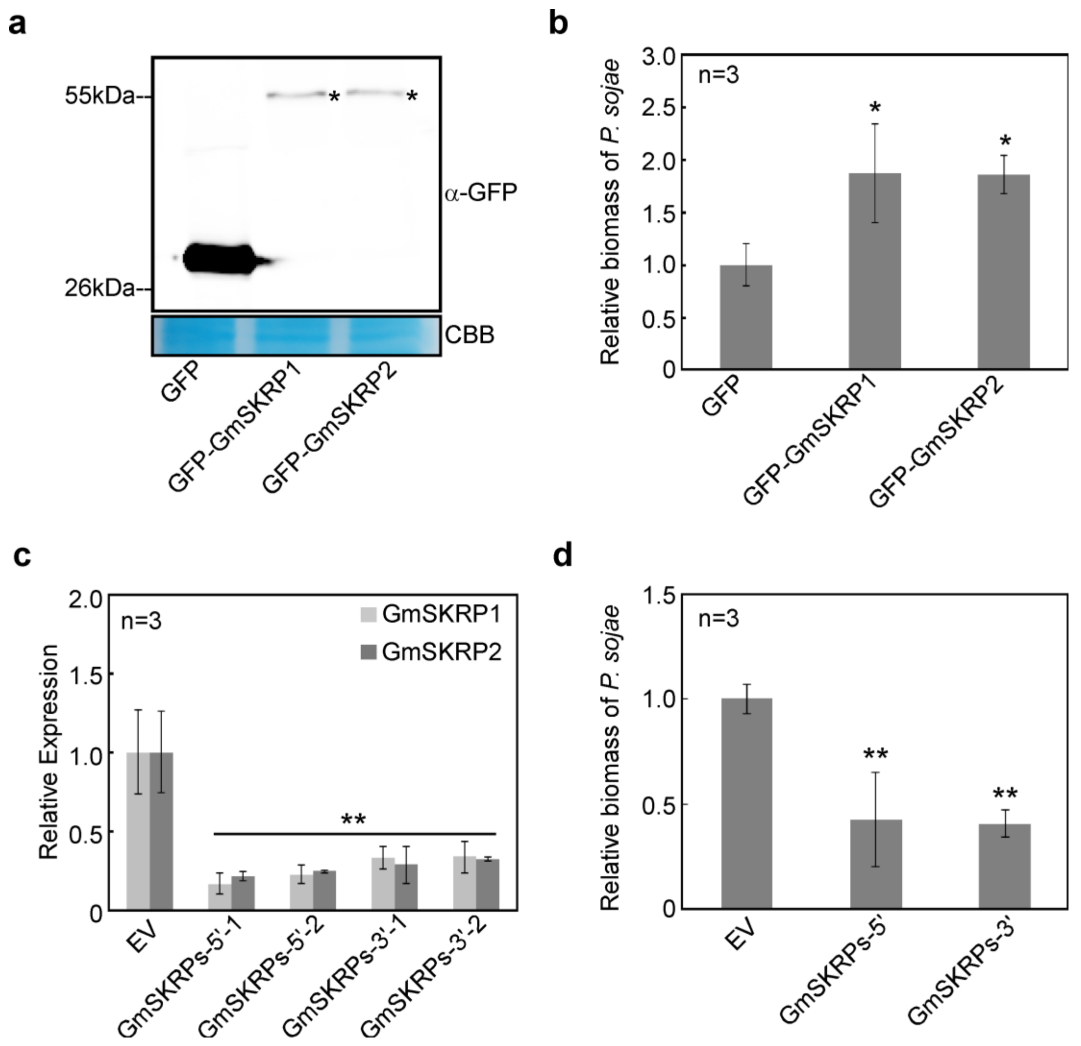

Supplementary Fig. 11. GmSKRP1/2 are negative regulators in soybean immunity against *P. sojae*.

(a) Immuno-blot analyses of the GFP-GmSKRP1/2 or GFP proteins in fluorescent hairy roots of soybean cv. Williams. Transformed hairy roots were selected based on the green fluorescence. The total protein was extracted from the hairy roots for analyses. Recombinant protein expression was confirmed by immuno-blotting using anti-GFP antibody. Protein bands are indicated by asterisks and the protein gel was stained with Coomassie Brilliant Blue (CBB) as the loading control.

(b) Relative biomass quantification of infected hairy roots over-expressing GFP-GmSKRP1/2 or GFP. Total DNA was extracted from inoculated hairy roots at 48 hpi and primers specific for soybean housekeeping gene *CYP2* and *P. sojae* actin gene were used for qRT-PCR. Means and standard errors from three replicates are shown (* $P<0.05$; one-way ANOVA). Three independent experiments gave similar results.

(c) Silencing efficiency of GmSKRPs genes in the fluorescent hairy roots. Transformed hairy roots were selected based on the red fluorescence. Total RNA was extracted from fluorescent hairy roots. The transcript levels of GmSKRP1/2 were measured by qRT-PCR, soybean housekeeping gene *CYP2* was used as an internal standard. Standard errors from three replicates are shown (** $P<0.01$; one-way ANOVA). Experiments were repeated three times with similar results.

(d) Relative biomass quantification of infected hairy roots. Total DNA was extracted from inoculated hairy roots at 48 hpi and primers specific for soybean housekeeping gene *CYP2* and *P. sojae* actin gene were used for qRT-PCR. Means and standard errors from three replicates are shown (** $P<0.01$; one-way ANOVA). Three independent experiments gave similar results.

Supplementary Fig. 12

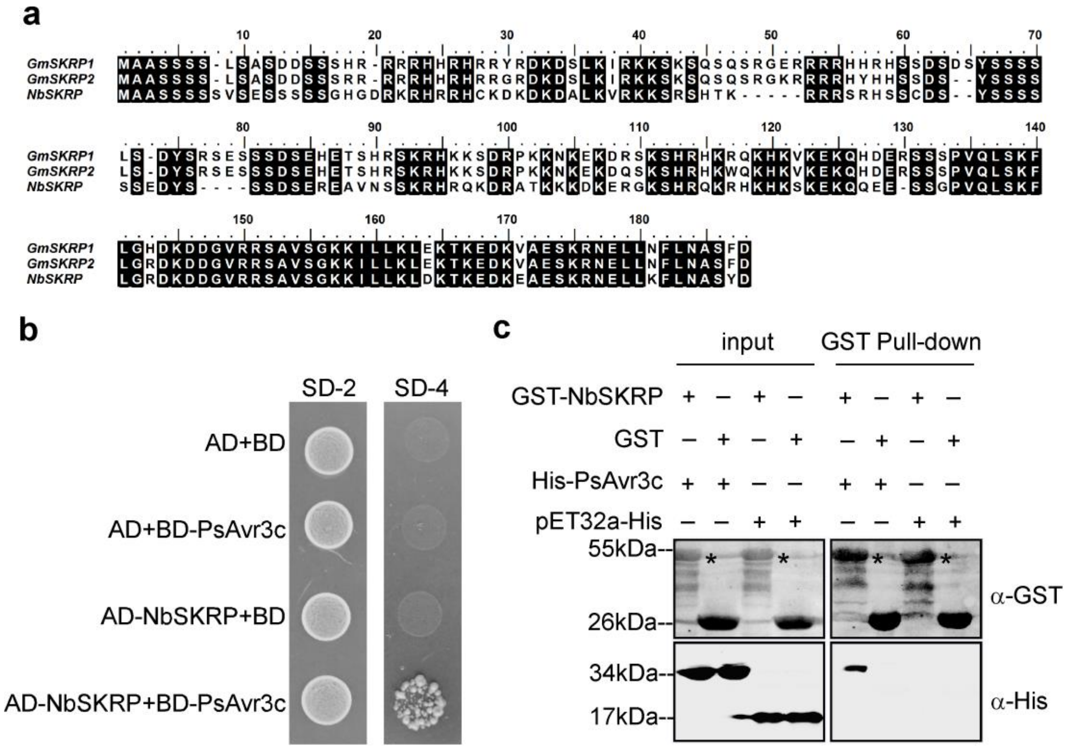

Supplementary Fig. 12. PsAvr3c interacts with *N. benthamiana* NbSKRP.

(a) Sequence alignment of *SKRP* homologous genes from soybean and *N. benthamiana*. The *NbSKRP* sequence (Niben101Scf00369g18022) is from *N. benthamiana* genome database (https://solgenomics.net/). BioEdit was used for the multiple alignment of the sequence.

(b) PsAvr3c interacts with NbSKRP in yeast. The *PsAvr3c* and *NbSKRP* genes were cloned into pGBKT7 and pGADT7, respectively. Yeast transformants were grown on SD/-Trp/-Leu (SD-2) and selected on SD/-Trp/-Leu/-His/-Ade (SD-4). The plates were photographed after 5 days.

(c) PsAvr3c interacts with NbSKRP *in vitro*. GST-NbSKRP or GST bound resins were incubated with supernatant from *E. coli* expressing His-PsAvr3c or pET32a-His empty vector. Protein bands of recombinant GST-NbSKRP are indicated by asterisks. The presence of His-PsAvr3c was detected by western blot using anti-His antibody.

Supplementary Fig. 13

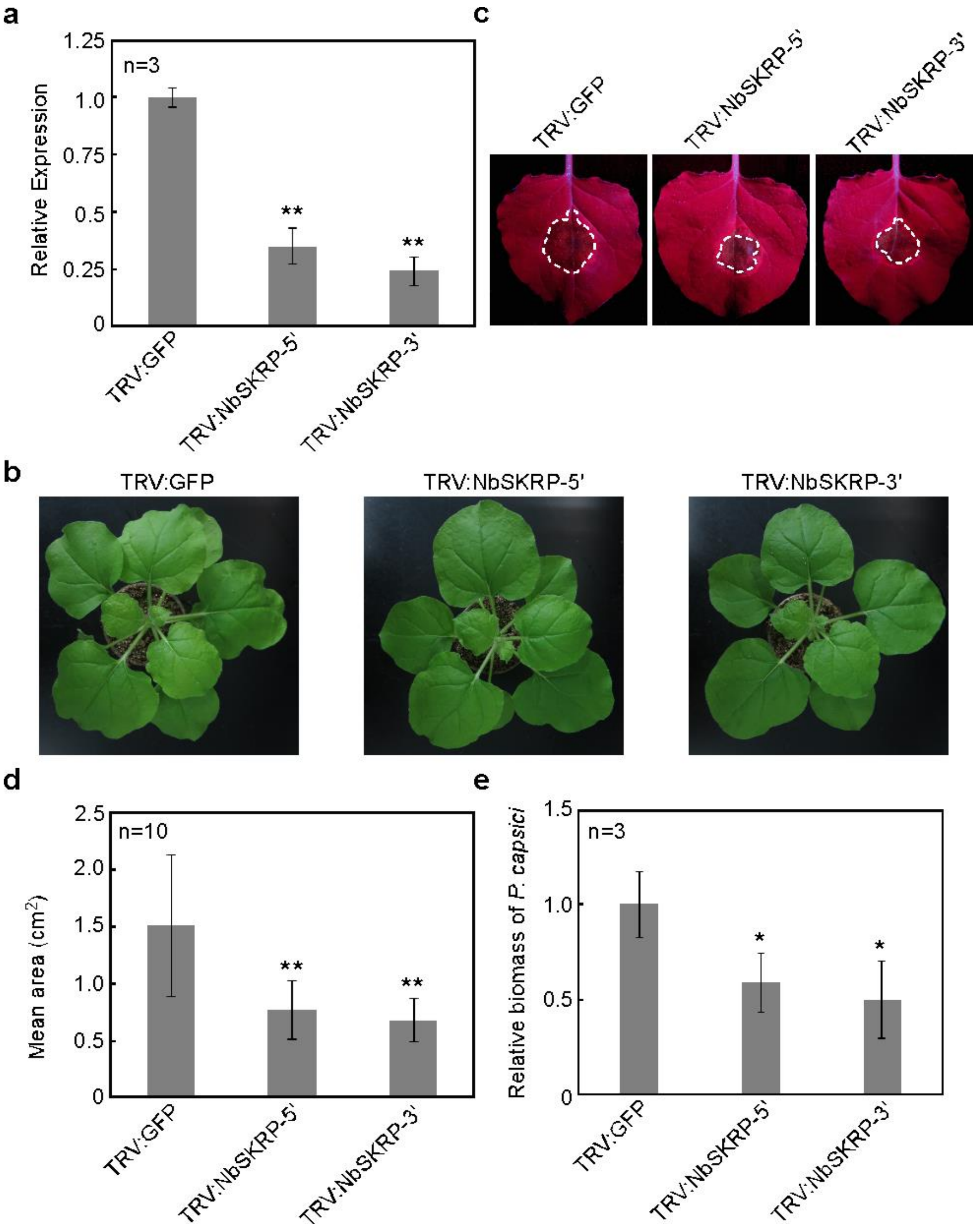

Supplementary Fig. 13. Silencing SKRP homologous gene in *N. benthamiana* enhances resistance to *P. capsici.*

(a) Silencing efficiency of *NbSKRP* gene in *N. benthamiana*. The qRT-PCR results show that *NbSKRP* is significantly silenced by virus induced gene silencing (VIGS) using fragments from 5' end and 3' end of *NbSKRP*. TRV-GFP was used as control. Means and standard errors from three replicates are shown. Asterisks indicate significant differences compared to TRV-GFP control based on one-way ANOVA (** $P<0.01$). Experiments were repeated three times with similar results.

(b) Silencing *NbSKRP* in *N. benthamiana* did not change the growth of plants. The images were taken at 30 days post-infiltration of TRV vectors.

(c) *P. capsici* infection assay on VIGS silenced *N. benthamiana* leaves. The dashed lines indicate the lesion areas. Representative photographs were taken at 30 hpi under UV light. The experiment was replicated three times with similar results.

(d) The average lesion areas of silenced *N. benthamiana* leaves inoculated with *P. capsici* mycellia. Measurements were taken at 30 hpi. Means and standard errors from ten replicates are shown. Asterisks indicate significant differences (** $P<0.01$; one-way ANOVA). The experiment was replicated three times with similar results.

(e) Relative biomass of *P. capsici* infected *N. benthamiana* leaves. DNA from *P. capsici* infected regions was isolated at 30 hpi for qPCR quantitative analyses. Means and standard errors from different replicates are shown (* $P<0.05$; one-way ANOVA). Three independent experiments gave similar results.

Supplementary Fig. 14

a

GmSKRP1 Candidate interactors	FLAG-GmSKRP1 IP			Control IP		
	No. of unique peptides			No. of unique peptides		
	Rep1	Rep2	Rep3	Rep1	Rep2	Rep3
NbSR45	5	4	1	0	0	0
NbRBP	3	1	1	0	0	0

b

unique peptides

>NbSR45
MAKPGRGRAASPSGSSSRSPSRSRSRSRSYSSRSSSSRSRSRSRSFSSSSSSGSSRS
PSPRPPPSQRKSPAGVSKRGRSPLPLSKKASPPPRKVSPTPESRVLHVDQLSRNVNE
NHLKEIFGNFGEILHVQLVIDHVVNLPKGFAYVEFKTRIDAEKAQLHMDGAQIDGKIVHA
KFTLPERKKAPSPPRAAATSSRRDAPRTDNAPVDLEKDGPKRQQELSPRRNPVSPPR
RSPIGRRGSPRREPDSPARRRAESPIRRRAGSPYRRGSPPAPRRRPASPIRRRSPSSP
PRRYRTPPRGSPRRIRGSPVRRRSPLPPRRRSPPRRARSPPRRSPVGRRSSRSPIRRP
IRSRSRSISPRRGRVPATRRGRSSSYSSSPSPRKAPRRISRSRSPRKPLRGRSPSNSDS
SSSPRKP

>NbRBP
MADSPRKRYSRSPSPWEKNSRSKSPPESYSRPRGRSRSRSRSRSRSRSRGRGEVSN
PGNTLYVTGLSTRVTERDLEEHFSKEGKVKSVFLVVEPRSRISRGFAFITMDSLEDANR
CIKHLNQSVLEGRYITVEKSRRKRARTPTPGHYLGLKNARGEGRGDRGRYRDREDYG
YRRSPRHSPYRSRRDYSPRRSPYGEGQEGSVLGRILLMQEAMLVVQDRSLSSPMNS

Supplementary Fig. 14. Mass spectrometry reveals GmSKRP1 associated with NbSR45 and NbRBP.

(a) Candidate proteins identified by mass spectrometry after co-immunoprecipitation of FLAG-GmSKRP1 and control. Peptide sequences matching a plant serine-arginine rich like protein SR45 (GenBank:AGB85016.1) and a predicted serine-arginine rich RNA binding protein (RBP) (GenBank:CAA70700.1) were consistently captured.

(b) Amino acid sequence features of candidate proteins from mass spectrometry. Unique peptides identified by mass spectrometry are highlighted in yellow.

Supplementary Fig. 15

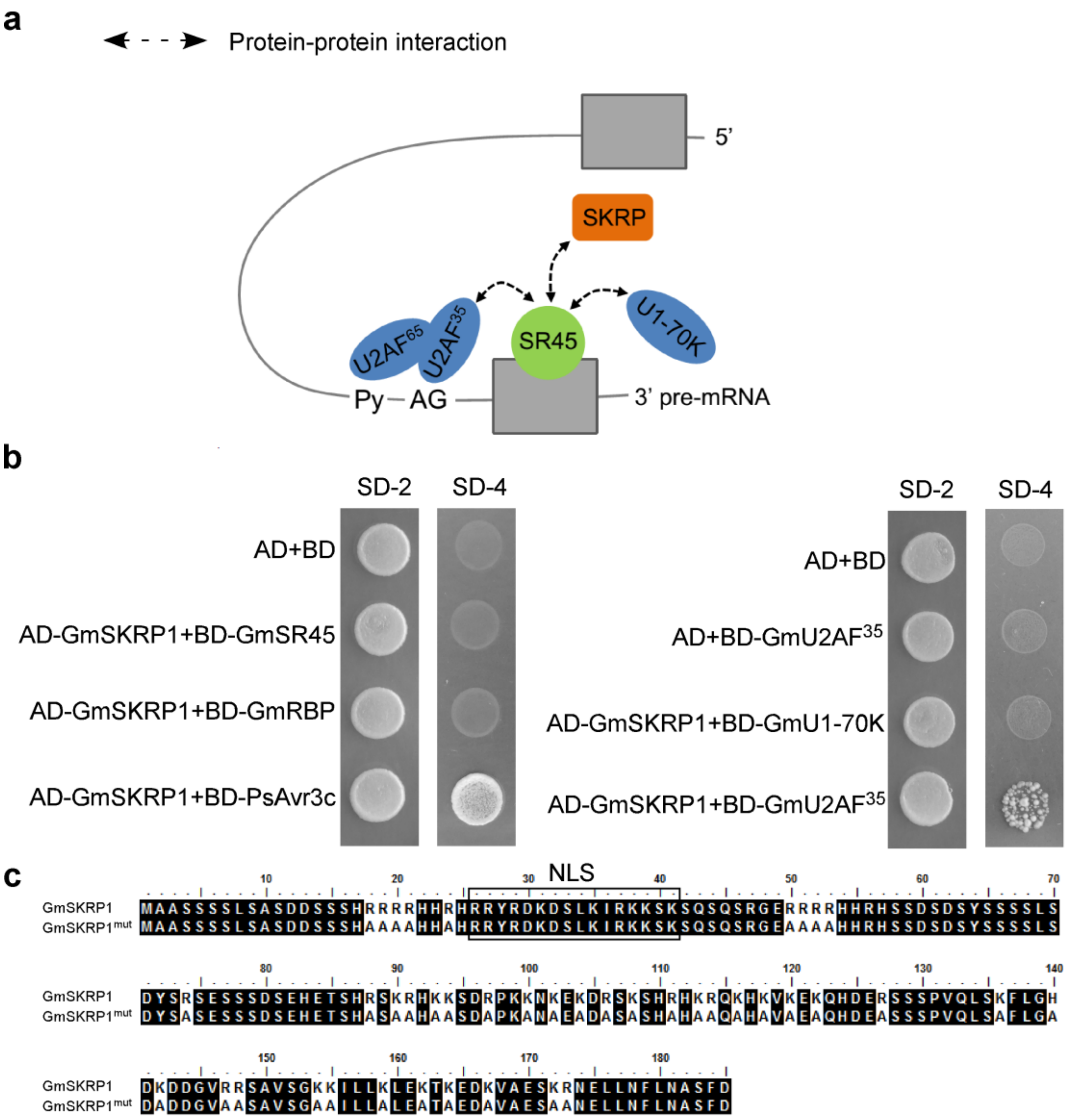

Supplementary Fig. 15. Verify the interaction between GmSKRP1 and plant spliceosome components in yeast.

(a) Schematic presentation of GmSKRP1 associates with splicesome components. SR45 associates with spliceosomal proteins U1-70K and U2AF35, these proteins form a complex *in vivo* and SKRP1 associates with this complex. This schematic is based on the results presented here and previously published data[2].

(b) GmSKRP1 interacts with GmU2AF35 in yeast. GmSR45, GmRBP, GmU1-70K, GmU2AF35, PsAvr3c (positive control) and GmSKRP1 were cloned into bait plasmid pGBKT7 (BD) and prey plasmid pGADT7 (AD), respectively. Yeast transformants were grown on SD/-Trp/-Leu (SD-2) and selected on SD/-Trp/-Leu/-His/-Ade (SD-4). The plates were photographed after 5 days.

(c) Amino acid sequence alignment of predicted proteins GmSKRP1 and GmSKRP1^{mut}. BioEdit was used for the multiple alignment of the sequence.

Supplementary Fig. 16

a

Sample ID	Left reads		Right reads	
	Input	Mapped	Input	Mapped
GFP-1	31816632	28733927 (90.3%)	31816632	27930759 (87.8%)
GFP-2	29417800	26427296 (89.8%)	29417800	25472140 (86.6%)
GFP-3	30769753	27660852 (89.9%)	30769753	26923132 (87.5%)
GmSKRP1-1	29376056	26865167(91.5%)	29376056	26125724 (88.9%)
GmSKRP1-2	32303696	29374229 (90.9%)	32303696	28464254 (88.1%)
GmSKRP1-3	21801601	19756994 (90.6%)	21801601	18875572 (86.6%)
PsAvr3c-1	23062183	20610802 (89.4%)	23062183	19901585 (86.3%)
PsAvr3c-2	22947369	20715140 (90.3%)	22947369	19901519 (86.7%)
PsAvr3c-3	24320167	21788972 (89.6%)	24320167	21063641 (86.6%)

b

Type		IR	ES	AD	AA
GmSKRP1-1 VS	GFP-1	1187/1091	516/436	443/402	913/829
	GFP-2	1167/1057	452/381	435/407	879/790
	GFP-3	1209/1086	559/456	478/440	903/816
GmSKRP1-2 VS	GFP-1	1222/1101	501/424	456/424	908/835
	GFP-2	1185/1077	498/411	428/393	931/834
	GFP-3	1184/1064	539/445	486/443	949/865
GmSKRP1-3 VS	GFP-1	1070/972	458/388	374/345	776/699
	GFP-2	1050/946	432/353	366/342	725/654
	GFP-3	1075/973	479/385	378/349	786/707
	overlap	745/629	263/201	175/157	510/435

c

Type		IR	ES	AD	AA
PsAvr3c-1 VS	GFP1	1557/1380	721/589	570/513	1002/902
	GFP2	1483/1329	601/494	538/495	947/862
	GFP3	1557/1385	646/527	568/514	1051/936
PsAvr3c-2 VS	GFP1	1564/1394	737/594	519/482	1002/905
	GFP2	1511/1357	627/507	532/486	934/847
	GFP3	1521/1366	704/567	546/497	959/859
PsAvr3c-3 VS	GFP1	1354/1238	620/498	441/408	814/739
	GFP2	1336/1209	530/434	431/394	802/727
	GFP3	13621238	612/490	477/440	818/731
	overlap	1300/1103	447/326	301/259	581/487

d

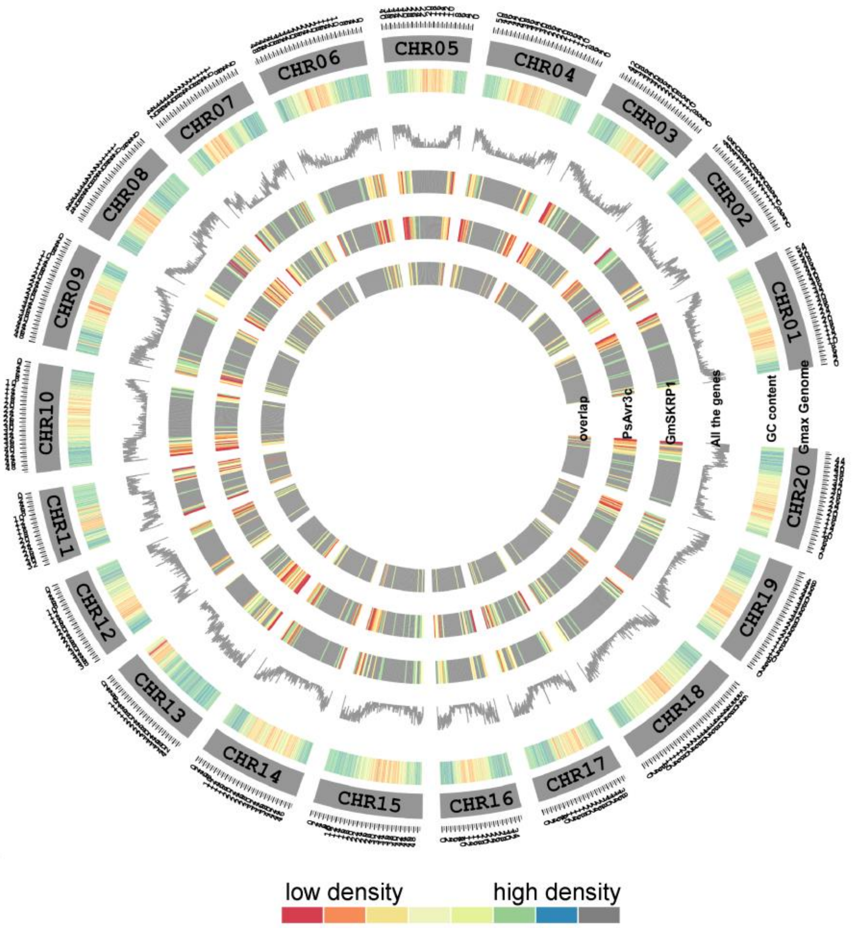

Supplementary Fig. 16. RNA splicing analyses based on RNA-seq data.

(a) Brief summary of RNA-seq data from soybean expressing GmSKRP1, PsAvr3c or GFP. Each treatment has three independent biological replicates. RNA-seq was conducted by llumina sequencing platform to produce pair-end reads with 150 bp length. The soybean genome was obtained from (ftp://ftp.jgi-psf.org/pub/compgen/phytozome/v9.0/early_release/Gmax_275_v 2.0) as reference genome. Reads mapping was performed by tophat-2.0.11 with anchor length more than 8nt for spliced alignments.

(b-c) Pairwise comparison of different RNA-seq data uncovers significant RNA alternative splicing events and corresponding gene numbers. The splicing difference was calculated based on the RNA-seq data from (a). The genes that are alternatively spliced at significant level between paired samples were categorized into four major AS types (IR: intron retention; AD: alternative donor site; ES: exon skipping; AA: alternative acceptor site). The number of overlapped genes was analyzed according to the web tool Venny (http://bioinfogp.cnb.csic.es/tools/venny/index.html).

(d) A Circos diagram with the two largest circles presented as a heat map and histogram of GC content and gene density in 100-kb windows, respectively; remaining three inner circles (from outer to inner) illustrate the heat map view of gene density including differential alternative splicing for GmSKRP1, PsAvr3c, and overlapped genes, respectively. Every colored line represents the genome coordinate of differential alternative splicing genes in 1000-kb windows and the line color indicated the density of differential alternative splicing genes. The red color represented lower density, while grey color referred to higher density. The color gradient has been shown at the bottom.

Supplementary Fig. 17

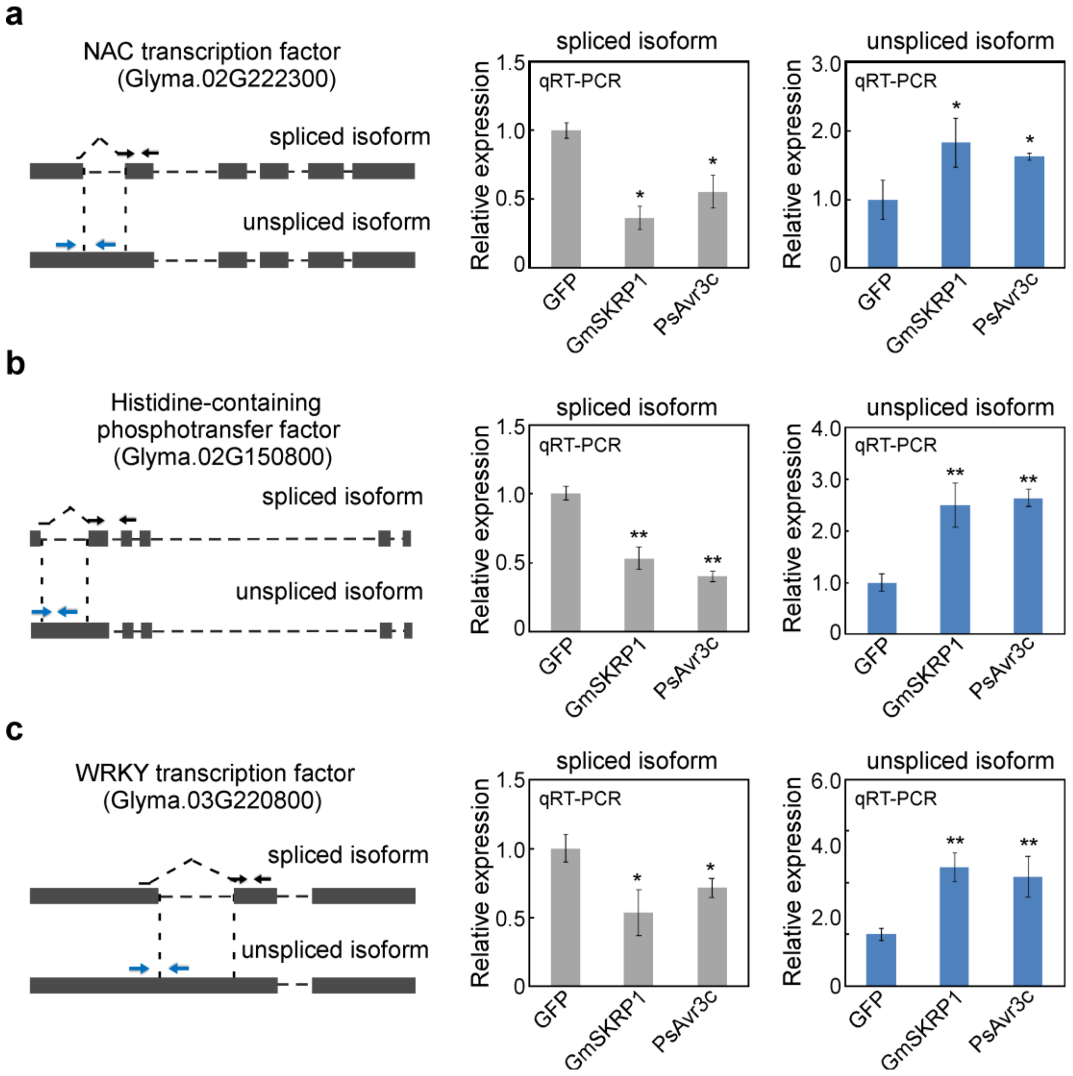

Supplementary Fig. 17. The relative fold change of the two isoforms caused by the AS were determined by qRT-PCR analyses.

(a-c) Analysis by qRT-PCR was performed using specific primers to measure different isoform transcript level of NAC transcription factor (a) and histidine-containing phosphotransfer factor (b) and WRKY transcription factor (c). The soybean actin gene *CYP2* was used as internal control gene. Means and standard errors from three replicates are shown (* $P<0.05$; ** $P<0.01$; one-way ANOVA). Experiments were repeated three times with similar results.

Supplementary Fig. 18

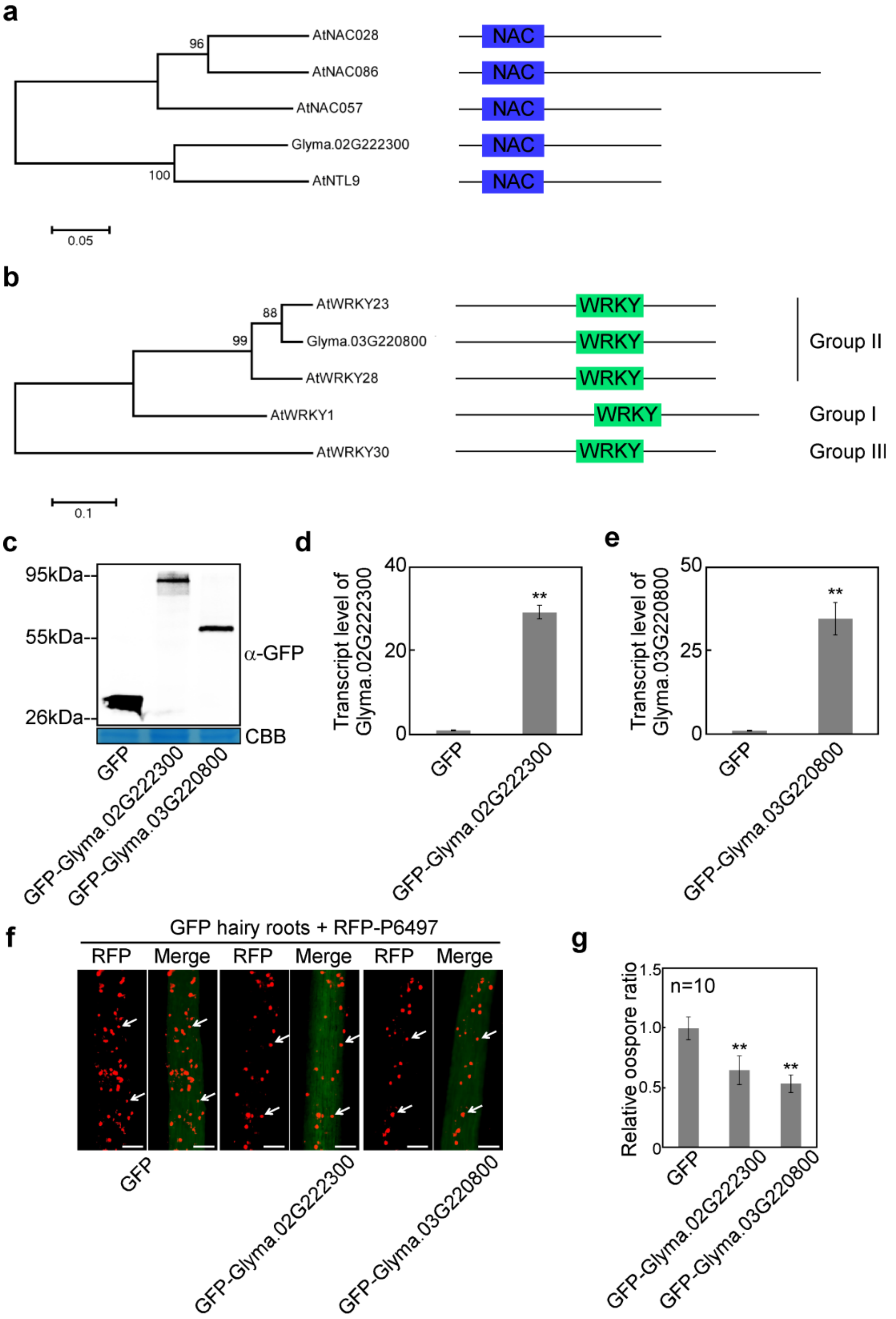

Supplementary Fig. 18. Phylogenetic analysis of NAC and WRKY transcription factors.

(a) Phylogenetic analysis of predicted Glyma.02G222300 and AtNAC transcription factors. Amino acid sequences (Supplementary Table 3) from NAC domain of different proteins are used for phylogenetic analysis. The tree shows that Glyma.02G222300 clusters together with NAC transcription factor NTL9. Schematic representation of full length NAC transcription factor proteins is shown on the right.

(b) Phylogenetic analysis of predicted Glyma.03G220800 and AtWRKY transcription factors. Amino acid sequences from the WRKY domain of the proteins are used for phylogenetic analysis. The tree shows that Glyma.03G220800 clusters together with AtWRKY transcription factor group II. AtWRKY28 is essential for SA synthesis activation, while AtWRKY23 is required for successful establishment of parasitic nematode feeding site. Schematic representation of full length WRKY transcription factors proteins is shown on the right.

(c) Immuno-blot analysis of GFP-Glyma.02G222300, GFP-Glyma.03G220800 and GFP in soybean (*rps3c*) hairy roots. The loading control gel was stained with Coomassie Brilliant Blue (CBB) to visualize protein.

(d-e) Total RNA from overexpressed samples were extracted. Analysis by qRT-PCR was performed using specific primers to measure transcript level of Glyma.02G222300 (d) and Glyma.03G220800 (e). The soybean actin gene CYP2 was used as internal control gene. Means and standard errors from three replicates are shown (** $P<0.01$; one-way ANOVA). Experiments were repeated three times with similar results.

(f) Over-expression of GFP-Glyma.02G222300 and GFP-Glyma.03G220800 in soybean (rps3c) hairy roots enhances soybean resistance. The hairy roots expressing GFP, GFP-Glyma.02G222300 or GFP-Glyma.03G220800 were inoculated with P. sojae strain P6497 expressing RFP. The P. sojae oospores were photographed at 48 hpi. The oospores were indicated with arrows. Three independent experiments gave similar results. Scale bars represent 0.25 mm.

(g) Statistics analysis of (df). Means and standard errors from ten replicates are shown. (** $P<0.01$; one-way ANOVA). Experiments were repeated three times with similar results.

Supplementary Fig. 19

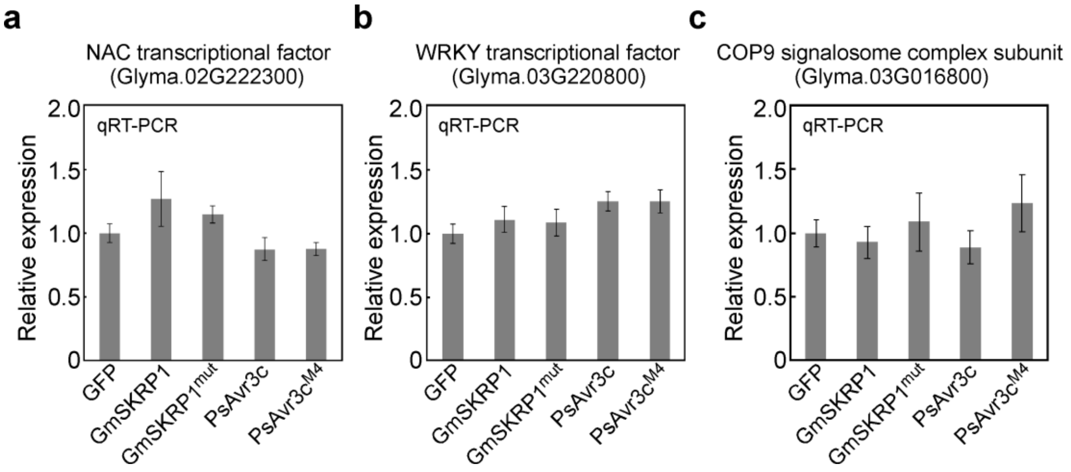

Supplementary Fig. 19. Selected defense related genes expression were determined by qRT-PCR analyses.

(a-c) Analysis by qRT-PCR was performed using specific primers to measure transcript level of NAC transcription factor (a) and WRKY transcription factor (b) and control gene COP9 signalosome complex subunit (c). The soybean actin gene *CYP2* was used as internal control gene. Means and standard errors from three biological replicates are shown. Experiments were repeated three times with similar results.

Supplementary Fig. 20

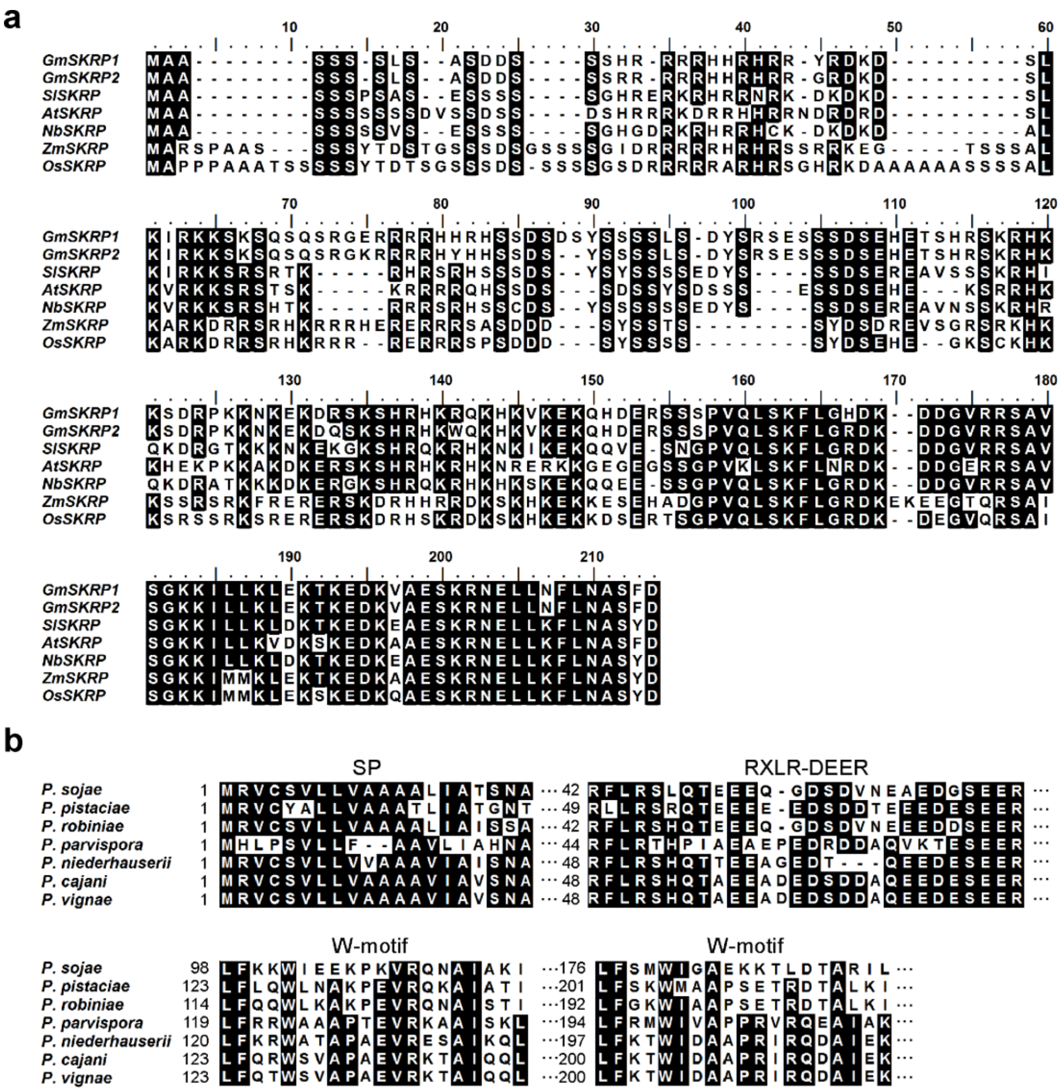

Supplementary Fig. 20. Partial alignment of predicted amino acid sequences of SKRP homologous proteins in plants and PsAvr3c homologous proteins in *Phytophthora* species.

(a) Sequence alignment of SKRP homologous proteins from soybean, tomato, *Arabidopsis*, *N. benthamiana*, zea mays, oryza sativa. The amino acid sequence (Supplementary Table 3) are from Phytozome database (https://phytozome.jgi.doe.gov/). BioEdit was used for the multiple alignment of the sequence.

(b) Partial sequence alignment of PsAvr3c-like proteins from species of *Phytophthora*. The amino acid sequence (Supplementary Table 3) alignment was generated by BioEdit. Identical residues are boxed in black.

Supplementary Fig. 21

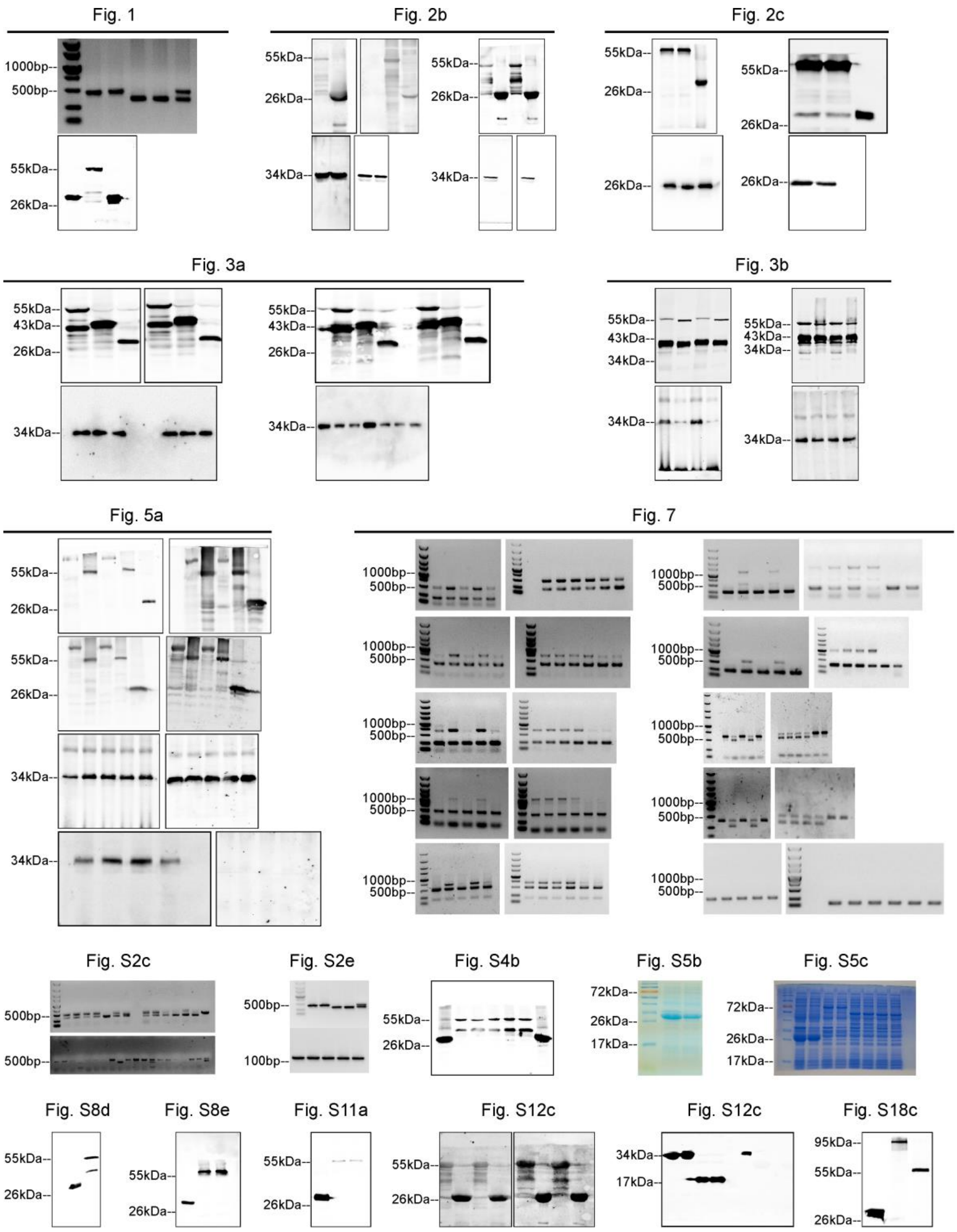

Supplementary Fig. 21. Original full length images for immunoblots and gels. The figures associated with each immunoblot or gel image was indicated above the images, molecular weight markers are indicated on the left.

Supplementary Table 1

Primer name	Primer sequence (5′ to 3′)
PsAvr3c-sgRNA1-F	CTAGCTAGCCGTCCTCTGATGAGTCCGTGAGGAC
PsAvr3c-sgRNA1-R	CTAGGGTCTCGAAACTATCGTATTTCTTGCGTCCT GACGAGCTTACTCGTTTCG
PsAvr3c-sgRNA2-F	CTAGCTAGCCCTGGACTGATGAGTCCGTGAGGAC
PsAvr3c-sgRNA2-R	CTAGGGTCTCGAAACTGTAGATGAGAGTTCCTGGA GACGAGCTTACTCGTTTCG
PsAvr3c-F	GAGAGGACTATTCGCCTGGA
PsAvr3c-R	TTGTGTTTCCTTCGGTATTG
qRT-PsojNIP-F	CCACGTCAACTGCGGGCG
qRT-PsojNIP-R	TTAAGCGTAGTAGGCGTTGC
qRT-PsXEG1-F	CACCAAGACCATCACCAA
qRT-PsXEG1-R	ATGAGGTACTGGCTGGAA
qRT-PsActin-F	ACTGCACCTTCCAGACCATC
qRT-PsActin-R	CCACCACCTTGATCTTCATG
GmCYP2-F	CGGGACCAGTGTGCTTCTTCA
GmCYP2-R	CCCCTCCACTACAAAGGCTCG
PC35-Actin-qRT-F	GTACTGCAACATCGTGCTGTCC
PC35-Actin-qRT-R	TTAGAAGCACTTGCGGTGCACG
NbActin-qRT-F	ACCATCAATGATCGGAATGGAA
NbActin-qRT-R	GCTCATCCTATCAGCAATGCC
GmSKRP1-qRT-F	GCCGATACCGTGATAAG
GmSKRP1-qRT-R	AGAGAGGGAAGATGAAGAA
GmSKRP2-qRT-F	ACTCTTCCGATTCCTATTC
GmSKRP2-qRT-R	ACTTATGACGGTGGCTTT
NbSKRP-qRT-F	ACTAAGTTTTTGGGGCGTGAT
NbSKRP-qRT-R	CTCTGCTTCCTTGTCCTCC
Glyma.02G222300-splicedF1	GACTTGCCTGATTTGTCAGT
Glyma.02G222300-splicedR1	GAGCACGACCGGTATAGA
Glyma.02G222300-unsplicedF2	ATGATGTTTGGGTTATTC
Glyma.02G222300-unsplicedR2	CCAATCCATCAACAGCAC
Glyma.02G222300-qRT-F	CATCTGCAAGCATTACAT
Glyma.02G222300-qRT-R	GCAATAGTTGACCATATTG
Glyma.03G220800-splicedF1	CAAAGGAACAGTTGAAGGCT
Glyma.03G220800-splicedR1	GGGCTGTTTTTCACAGCTT
Glyma.03G220800-unsplicedF2	TCCGAACTCTTCATCCAT
Glyma.03G220800-unsplicedR2	ATGCCATGAGCCATTCACA
Glyma.03G220800-qRT-F	AGCAGCATCAACAAGTCCACATCG
Glyma.03G220800-qRT-R	ATCTTCTTTCAACATATGTGAAGA
Glyma.03G016800-splicedF1	AGAGTTAAAGAGTAAAGTTA

Glyma.03G016800-splicedR1	CTTGAGTAAAAATCATTTATC
Glyma.03G016800-unsplicedF2	AACAGGACCTGAACTGGGAA
Glyma.03G016800-unsplicedR2	CTGCCAGATATTGAAAAAG
Glyma.03G016800-qRT-F	ACAAACTTAATTAAGGAAAGCA
Glyma.03G016800-qRT-R	GCACTCATACACATATGAACAAT
Glyma.02G150800-splicedF1	GTTTCAACAGGGTTTTTTGG
Glyma.02G150800-splicedR1	GATGAGCATGAGCATCTATT
Glyma.02G150800-unsplicedF2	GTTTCAACAGGTTCTCTTGT
Glyma.02G150800-unsplicedR2	ACCTTGCAGTCCAAGAGTCT
BD-PsAvr3c-F	GGAGGCCGAATTCCCGATGGTTGAGCCTTCCGCCAC
BD-PsAvr3c-R	GGTCGACGGATCCCCTTACTTGTGTTTCCTTC
AD-GmSKRPs-ECORI F	CCGGAATTCATGGCGGCCTCTTCTTCCT
AD-GmSKRPs-BamHI R	CGCGGATCCCTAATCAAAACTAGCATTTAAAAAG
AD-NbSKRP-F	CAGTGAATTCCACCCGATGGCCGCCTCATCGTCC
AD-NbSKRP-R	GTATCGATGCCCACCCTCAATCATAACTTGCATTC
PGEX4T-2-GmSKRPs-F	TCCCCAGGAATTCCCATGGCGGCCTCTTCTTCCT
PGEX4T-2-GmSKRPs-R	CGCTCGAGTCGACCCCTAATCAAAACTAGCATTTAAAAAG
PGEX4T-2-NbSKRP-F	TCCCCAGGAATTCCCATGGCCGCCTCATCGTCC
PGEX4T-2-NbSKRP-R	CGCTCGAGTCGACCCTCAATCATAACTTGCATTC
pET32a-PsAvr3c-F	CCGGAATTCCTATTCGCCTGGATCAAAAACGC
pET32a-PsAvr3c-R	ATAAGAATGCGGCCGCTTACTTGTGTTTCCTTCGG
PBinGFP-PsAvr3c-F	TACAAGGGTACCCCCATGGTTGAGCCTTCCGCCAC
PBinGFP-PsAvr3c-R	GGATCCGTCGACCCCTTACTTGTGTTTCCTTC
PBinGFP-PsAvr3c(-nNLS)-NESR	GGATCCGTCGACCCCTTAAAGAGTAAGTCTTTCAAGAGGA GGAAGTTGAAGCATATACTCTTTGGCCGGC
PBinGFP-PsAvr3c(-nNLS)-NLSR	GGATCCGTCGACCCCCTATCCTCCAACCTTTCTCTTCTTC TTAGGCTGATACTCTTTGGCCGGC
PBinGFP-PsAvr3c(-nNLS)-nlsR	GGATCCGTCGACCCCAAGGGTCCTCAACCTTACGCTTGTT TTTAGGATACTCTTTGGCCGGC
PBinGFP-PsAvr3c(-nNLS)-R	GGATCCGTCGACCCCCTAATACTCTTTGGCCGGC
PENTY-NRFP-GmSKRPs-F	CACCATGGCGGCCTCTTCTTCCT
PENTY-NRFP-GmSKRPs-R	CTAATCAAAACTAGCATTTAAAAAG
pICH86988-GmSKRPs-FLAG-F	TTTGGTCTCAAATGGATTACAAGGATGACGACGATAAGAT GGCGGCCTCTTCTTCCTC
pICH86988-GmSKRPs-R	TTTGGTCTCCAAGCCTAATCAAAACTAGCATTTAAAAAG
PBinGFP-Glyma.16G121300-F	TACAAGGGTACCCCCATGGCTGATTCTCCCCTTGC
PBinGFP-Glyma.16G121300-R	GGATCCGTCGACCCCTCACCTAGATCCACGAGCA
PBinGFP-Glyma.15G255400-F	TACAAGGGTACCCCCATGGCGAAACCTGGGCGAGG
PBinGFP-Glyma.15G255400-R	GGATCCGTCGACCCCCTATGGCTTGCGAGTTGGTG
PBinGFP-Glyma.07G159900-F	TACAAGGGTACCCCCATGGCGGAGCACCTTGCAT
PBinGFP-Glyma.07G159900-R	GGATCCGTCGACCCCTCATTGTTTCTCTTCCCTC
PBinGFP-Glyma.01G175100-F	TACAAGGGTACCCCCATGGGGGACAACAACAGCAA

PBinGFP-Glyma.01G175100-R	GGATCCGTCGACCCCCTAATAATCATACTCCCGGGA
PBinGFP-03G220800-F	TACAAGGGTACCCCCATGGAGAATACTAAGATGATG
PBinGFP-03G220800-R	GGATCCGTCGACCCCCTAATCTTCTTTCAACATATG
PBinGFP-02G222300-F	TACAAGGGTACCCCCATGGGTGCCGTCGTCGAGTGTTA
PBinGFP-02G222300-R	GGATCCGTCGACCCCTTAAGATCTGACATATGCCCAT
PBinGFP-F	AAGACCCCAACGAGAAGC
PBinGFP-R	GAACCCTAATTCCCTTATCTG
pet32a-F	TAATACGACTCACTATAGGG
pet32a-R	GCTAGTTATTGCTCAGCGG
pGADT7	TAATACGACTCACTATAGgg
3'AD	AGATGGTGCACGATGCACAG
3'BD	TAAGAGTCACTTTAAAATTTGTAT
pGEX 3'R	CCGGGAGCTGCATGTGTCAGAGG
pGEX 5'F	GGGCTGGCAAGCCACGTTTGGTG
M13F(-47)	CGCCAGGGTTTTCCCAGTCACGAC
M13R(-48)	AGCGGATAACAATTTCACACAGGA
pICH86988-F	GGACACGCTCGAGTATAAGAGCTC
pICH86988-R	GGATCTGAGCTACACATGCTCAGG
TRV-NbSKRP-5'-F	CGACAAGACCCTGCAAAAATTCAAGTAGCGTGT
TRV-NbSKRP-5'-R	GAGAAGAGCCCTGCACAGAGGAAGAGGAAGAAGA
TRV-NbSKRP-3'-F	CGACAAGACCCTGCAATGGCCGCCTCATCGTCCT
TRV-NbSKRP-3'-R	GAGAAGAGCCCTGCAGGCTCTATCTTTCTGCCT
GmSKRPs-5’-F	CACCATGGCGGCCTCTTCTTC
GmSKRPs-5’-R	ATGCCTACGGCGTCGTT
GmSKRPs-3’-F	CACCTATTCTTCATCTTCCCTCT
GmSKRPs-3’-R	TTTTCCTTATTCTTCTTT
RT-Glyma.01G238400-F	TTACTCAGGCACTGTAGCTGG
RT-Glyma.01G238400-R	TAGGAACATTGTTTGGCACG
RT-Glyma.02G150800-F	AAGGGGTGTCCGAGTTTTGAA
RT-Glyma.02G150800-R	CGTGCCATGTTTTTTAGGAG
RT-Glyma.02G222300-F	ATGATGTTTGGGTTATTC
RT-Glyma.02G222300-R	GAGCACGACCGGTATAGA
RT-Glyma.03G099900-F	TTGGCAACAAAAGGCAGCAA
RT-Glyma.03G099900-R	TTGGATCTGGCAACCCAAT
RT-Glyma.03G220800-F	AAGATGATGGGGGTGAAGAT
RT-Glyma.03G220800-R	TGGGAAAGGGGCTGTTTTT
RT-Glyma.09G203500-F	CATCCCTGGCATCACTGACTA
RT-Glyma.09G203500-R	TTTCCCCACCAAAGGTCGTT
RT-Glyma.16G073600-F	TCCATATCCCCGTTTTCCTT
RT-Glyma.16G073600-R	TTCCAACTCCACCAGCACCT
RT-Glyma.20G228900-F	ATTCATTACGAGACAACGGGCC
RT-Glyma.20G228900-R	CTGAATAACTTCATCTAGGAGAT

RT-Glyma.19G108300-F	ATAGCGACGAGACCAGCGA
RT-Glyma.19G108300-R	GAGACAGGTCACGGCTAGAT
RT-Glyma.05G230000-F	ATGGCATCATTGAAGGTAAT
RT-Glyma.05G230000-R	TCATAAAGGCATGGAAATAC

Supplementary Table 1. Primers used in this study.

Supplementary Table 2

Locus	AS region	Type	P Value	Function description
Glyma.09G203500	Chr09:42792555-42792829	IntronR	8.88178E-16	phosphoenolpyruvate carboxykinase 1
Glyma.02G109400	Chr02:10495593-10496102	IntronR	1.08802E-14	cAMP-regulated phosphoprotein 19-related protein
Glyma.15G262500	Chr15:49520546-49520925	IntronR	5.73985E-14	cysteine synthase D1
Glyma.13G187200	Chr13:30107690-30108665	IntronR	8.72635E-14	Proteasome component (PCI) domain protein
Glyma.10G284200	Chr10:50452877-50453170	IntronR	9.17044E-14	Ataxia telangiectasia-mutated and RAD3-related
Glyma.17G036700	Chr17:2682503-2683315	IntronR	1.14686E-13	Rab5-interacting family protein
Glyma.20G168400	Chr20:40614932-40615395	IntronR	2.02616E-13	polypyrimidine tract-binding protein 3
Glyma.12G032800	Chr12:2480457-2480774	IntronR	4.27602E-12	ubiquitin-conjugating enzyme 10
Glyma.13G316100	Chr13:41089578-41090217	IntronR	7.76923E-12	Calcium-binding EF-hand family protein
Glyma.19G231600	Chr19:48212716-48213267	IntronR	1.78531E-11	GmSKRP2
Glyma.16G129000	Chr16:28117730-28118938	IntronR	6.71464E-10	zinc finger (Ran-binding) family protein
Glyma.02G087000	Chr02:7563164-7563374	IntronR	6.96004E-10	CHASE domain containing histidine kinase protein
Glyma.14G220000	Chr14:48507349-48507799	IntronR	1.01174E-09	Calcium-dependent lipid-binding family protein
Glyma.06G055100	Chr06:4163548-4164261	IntronR	1.35924E-09	apolipoprotein N-acyltransferase family protein
Glyma.06G276900	Chr06:46815544-46815759	IntronR	4.13553E-09	Mitogen activated protein kinase
Glyma.20G228900	Chr20:46304627-46305533	IntronR	1.11506E-08	cysteine synthase D1
Glyma.06G324000	Chr06:51173647-51173935	IntronR	1.60949E-08	U2 snRNP auxilliary factor
Glyma.05G130300	Chr05:32322456-32322931	IntronR	8.69521E-08	DNA-directed RNA polymerase II
Glyma.12G169700	Chr12:32450722-32452346	IntronR	2.2241E-07	transmembrane nine 1
Glyma.02G087000	Chr02:7560170-7560880	IntronR	3.78228E-07	CHASE domain containing histidine kinase protein
Glyma.02G150800	Chr02:15527733-15528090	IntronR	4.75263E-07	histidine-containing phosphotransfer factor 5
Glyma.12G111000	Chr12:10601338-10601766	IntronR	1.36996E-06	GCR2-like 2
Glyma.20G241400	Chr20:47242097-47242451	IntronR	2.68337E-06	structural maintenance of chromosomes protein
Glyma.16G210400	Chr16:36950070-36950930	IntronR	4.01601E-06	ankyrin repeat protein
Glyma.01G138300	Chr01:46563782-46564018	IntronR	4.24485E-06	methionine aminopeptidase 1A
Glyma.14G155700	Chr14:33848627-33848742	IntronR	5.98557E-06	GDSL-like Lipase/Acylhydrolase superfamily protein
Glyma.20G168400	Chr20:40611811-40612127	IntronR	1.15205E-05	polypyrimidine tract-binding protein 3
Glyma.12G005700	Chr12:434311-435287	IntronR	1.27813E-05	autophagy 3 (APG3)
Glyma.09G184700	Chr09:40996380-40997031	IntronR	1.48392E-05	DNAse I-like superfamily protein
Glyma.17G075200	Chr17:5904481-5904580	IntronR	1.99906E-05	LJRHL1-like 1
Glyma.08G210900	Chr08:17032848-17033838	IntronR	2.76261E-05	peptide deformylase 1A
Glyma.18G020900	Chr18:1530121-1530921	IntronR	3.65289E-05	bZIP transcription factor family protein
Glyma.02G144300	Chr02:14886173-14886305	IntronR	4.66736E-05	2-oxoglutarate and Fe(II)-dependent oxygenase protein
Glyma.17G259400	Chr17:41353077-41353225	IntronR	5.03418E-05	RING/FYVE/PHD zinc finger superfamily protein
Glyma.20G123800	Chr20:36647004-36648551	IntronR	5.92926E-05	FAM133-like protein
Glyma.04G215400	Chr04:48691849-48692298	IntronR	6.58373E-05	TLD-domain containing nucleolar protein
Glyma.04G255700	Chr04:52209694-52209980	IntronR	6.76191E-05	U2 snRNP auxilliary factor
Glyma.13G163500	Chr13:27877629-27877723	IntronR	7.04317E-05	Leucine-rich repeat family protein
Glyma.08G245100	Chr08:21174261-21175708	IntronR	7.19007E-05	RS2-interacting KH protein

Glyma.13G244500	Chr13:35389249-35389920	IntronR	7.29562E-05	Protein of unknown function (DUF707)
Glyma.06G069200	Chr06:5298035-5298657	IntronR	8.06421E-05	DEAD/DEAH box RNA helicase family protein
Glyma.03G158400	Chr03:37369666-37369954	IntronR	8.58373E-05	Aldolase-type TIM barrel family protein
Glyma.18G250700	Chr18:53704929-53705706	IntronR	0.000114399	Subtilisin-like serine endopeptidase protein
Glyma.04G200100	Chr04:47265264-47265633	IntronR	0.000125847	coiled-coil protein
Glyma.08G040200	Chr08:3168557-3168823	IntronR	0.000131761	DNAse I-like superfamily protein
Glyma.14G009100	Chr14:700293-700765	IntronR	0.000198162	chloroplast / phytochromobilin synthase (HY2)
Glyma.01G162200	Chr01:50028810-50029199	IntronR	0.000231313	RNA polymerase II large subunit
Glyma.20G168400	Chr20:40614821-40614917	IntronR	0.000258347	polypyrimidine tract-binding protein 3
Glyma.04G066200	Chr04:5512822-5512903	IntronR	0.000280065	Nucleotide/sugar transporter family protein
Glyma.03G220800	Chr03:42405821-42406211	IntronR	0.000302293	WRKY DNA-binding protein 23
Glyma.12G084200	Chr12:6735013-6735192	IntronR	0.00030309	alpha-adaptin
Glyma.09G116600	Chr09:27022587-27022755	IntronR	0.000312803	Chaperone DnaJ-domain superfamily protein
Glyma.19G136600	Chr19:39788394-39788623	IntronR	0.000317907	tRNA-splicing endonuclease subunit
Glyma.18G206500	Chr18:49029274-49029384	IntronR	0.000327963	MATE efflux family protein
Glyma.14G064900	Chr14:5332449-5332778	IntronR	0.000337183	Mitochondrial substrate carrier family protein
Glyma.04G204500	Chr04:47688627-47688913	IntronR	0.000361578	Protein prenyltransferase superfamily protein
Glyma.02G000100	Chr02:16061-16491	IntronR	0.000402019	B-block binding subunit of TFIIIC
Glyma.05G062900	Chr05:5981665-5981907	IntronR	0.000428834	Phosphotyrosine protein phosphatases protein
Glyma.04G195600	Chr04:46737572-46738087	IntronR	0.00043563	purple acid phosphatase 27
Glyma.13G150200	Chr13:26367020-26367092	IntronR	0.000444597	KDO transferase A
Glyma.11G016200	Chr11:1144143-1144559	IntronR	0.000449677	cytochrome P450, subfamily B, polypeptide 3
Glyma.02G095200	Chr02:8541756-8542037	IntronR	0.000528301	beta-hexosaminidase 1
Glyma.02G144300	Chr02:14887778-14887872	IntronR	0.000530592	2-oxoglutarate and Fe(II)-dependent oxygenase protein
Glyma.09G136800	Chr09:33865931-33866284	IntronR	0.000551454	oxidoreductase metal ion-binding protein
Glyma.08G188400	Chr08:15115732-15117090	IntronR	0.000582207	transmembrane nine 1
Glyma.06G092800	Chr06:7313994-7314335	IntronR	0.000585151	Rhamnogalacturonate lyase family protein
Glyma.04G112700	Chr04:12643510-12643712	IntronR	0.000588917	Transducin/WD40 repeat-like superfamily protein
Glyma.13G118800	Chr13:23155034-23155108	IntronR	0.000593717	P-glycoprotein 11
Glyma.15G033500	Chr15:2666166-2666300	IntronR	0.000597431	ZIP metal ion transporter family
Glyma.01G073000	Chr01:13462027-13462725	IntronR	0.000647112	FAD-linked oxidases family protein
Glyma.06G093100	Chr06:7333154-7333235	IntronR	0.000689626	phosphoglucose isomerase 1
Glyma.07G167300	Chr07:27016415-27016669	IntronR	0.000751934	Zinc-binding dehydrogenase family protein
Glyma.01G217100	Chr01:54746481-54746560	IntronR	0.000826532	PAS domain-containing tyrosine kinase protein
Glyma.02G144300	Chr02:14885871-14886052	IntronR	0.000834549	2-oxoglutarate and Fe(II)-dependent oxygenase protein
Glyma.11G038800	Chr11:2769927-2770694	IntronR	0.000916401	Protein kinase superfamily protein
Glyma.02G222300	Chr02:40991832-40992273	IntronR	0.000928488	NAC transcription factor-like 9
Glyma.19G047800	Chr19:7485555-7486317	IntronR	0.000939699	abscisic acid (aba)-deficient 4
Glyma.16G058500	Chr16:5730235-5730640	IntronR	0.000953567	hypothetical protein
Glyma.06G130300	Chr06:10715397-10715531	IntronR	0.000986649	Tetratricopeptide repeat (TPR)-like superfamily protein
Glyma.08G319000	Chr08:43815856-43816271	IntronR	0.001048824	zinc finger (C2H2 type) family protein
Glyma.13G187200	Chr13:30107690-30108648	IntronR	0.001062418	Proteasome component (PCI) domain protein

Glyma.03G264600	Chr03:45751459-45752183	IntronR	0.00107223	RNA-binding protein
Glyma.05G003500	Chr05:271612-272023	IntronR	0.001156752	embryo defective 3012
Glyma.17G259400	Chr17:41357516-41357650	IntronR	0.001172699	RING/FYVE/PHD zinc finger superfamily protein
Glyma.08G213400	Chr08:17225725-17225852	IntronR	0.0012226	Molecular chaperone Hsp40/DnaJ family protein
Glyma.08G339700	Chr08:45600151-45600268	IntronR	0.001281933	nodulin MtN21 /EamA-like transporter protein
Glyma.17G097500	Chr17:7668849-7669164	IntronR	0.001289745	P-loop containing nucleoside triphosphate hydrolases
Glyma.05G241600	Chr05:41658677-41658901	IntronR	0.001345128	CHASE domain containing histidine kinase protein
Glyma.12G159000	Chr12:27385970-27386068	IntronR	0.001451843	O-fucosyltransferase family protein
Glyma.08G220500	Chr08:17914445-17914535	IntronR	0.001465135	ATP-dependent DNA helicase
Glyma.17G173200	Chr17:17895979-17896744	IntronR	0.001489277	dihydroflavonol 4-reductase
Glyma.07G102300	Chr07:9750762-9750856	IntronR	0.001494068	FAD/NAD(P)-binding oxidoreductase protein
Glyma.08G111200	Chr08:8560838-8561607	IntronR	0.001536147	RAB geranylgeranyl transferase beta subunit 1
Glyma.07G060400	Chr07:5361657-5361865	IntronR	0.001570976	G-box binding factor 3
Glyma.16G058200	Chr16:5708774-5709005	IntronR	0.00157491	uracil phosphoribosyltransferase 1
Glyma.17G056900	Chr17:4308274-4308650	IntronR	0.001577263	Protein kinase superfamily protein
Glyma.10G048700	Chr10:4385241-4385468	IntronR	0.001626926	RNA binding family protein
Glyma.16G080900	Chr16:8640359-8641713	IntronR	0.001634284	beta-hydroxyisobutyryl-CoA hydrolase 1
Glyma.03G256800	Chr03:45163159-45163585	IntronR	0.00175444	Glycosyl hydrolase superfamily protein
Glyma.07G048000	Chr07:4028754-4029031	IntronR	0.001819297	NAC transcription factor-like 9
Glyma.07G122300	Chr07:14316436-14316530	IntronR	0.001873156	tRNA (guanine-N-7) methyltransferase
Glyma.14G064900	Chr14:5332848-5334012	IntronR	0.001975941	Mitochondrial substrate carrier family protein
Glyma.07G001600	Chr07:158029-158108	IntronR	0.002050281	Tetratricopeptide repeat (TPR)-like superfamily protein
Glyma.03G210900	Chr03:41715887-41716003	IntronR	0.002118621	catalytics
Glyma.14G128800	Chr14:21569640-21570368	IntronR	0.002148043	mitotic-like cyclin 3B from Arabidopsis
Glyma.12G200100	Chr12:36147283-36147459	IntronR	0.002270823	diacylglycerol kinase 5
Glyma.01G138300	Chr01:46567713-46568110	IntronR	0.002321046	methionine aminopeptidase 1A
Glyma.10G047800	Chr10:4299126-4300079	IntronR	0.002421786	Ypt/Rab-GAP domain of gyp1p superfamily protein
Glyma.07G198600	Chr07:36704677-36705262	IntronR	0.002486624	Ribosomal protein S6e
Glyma.08G003100	Chr08:243350-243447	IntronR	0.002554303	fatty acid amide hydrolase
Glyma.15G047400	Chr15:3756775-3756857	IntronR	0.002834465	RHOMBOID-like protein 10
Glyma.04G232900	Chr04:50124780-50124986	IntronR	0.002950285	PUA domain-containing protein
Glyma.07G199900	Chr07:36855906-36856133	IntronR	0.003017533	polyubiquitin 10
Glyma.02G024500	Chr02:2181213-2181335	IntronR	0.003093447	Tetratricopeptide repeat (TPR)-like superfamily protein
Glyma.14G223500	Chr14:48878246-48878401	IntronR	0.003369416	catalase 2
Glyma.04G068900	Chr04:5759767-5759868	IntronR	0.003378873	no annotation
Glyma.08G245100	Chr08:21174261-21176427	IntronR	0.003561429	RS2-interacting KH protein
Glyma.06G030100	Chr06:2364253-2366078	IntronR	0.003640943	Inositol-pentakisphosphate 2-kinase family protein
Glyma.19G047800	Chr19:7487058-7487140	IntronR	0.003704059	abscisic acid (aba)-deficient 4
Glyma.09G161500	Chr09:38582048-38583308	IntronR	0.003705968	no annotation
Glyma.08G210900	Chr08:17032375-17032566	IntronR	0.003760243	peptide deformylase 1A
Glyma.11G253100	Chr11:34401294-34401495	IntronR	0.003812543	DNA topoisomerase, type IA, core
Glyma.08G030000	Chr08:2398445-2398650	IntronR	0.003848522	Metallopeptidase M24 family protein

Glyma.20G193600	Chr20:43239615-43239885	IntronR	0.003977049	Homeodomain-like superfamily protein
Glyma.05G064100	Chr05:6244920-6245144	IntronR	0.004034318	calcium-dependent protein kinase 29
Glyma.20G117100	Chr20:35983789-35983915	IntronR	0.004076583	glutamine-tRNA ligase
Glyma.14G093200	Chr14:8663924-8666093	IntronR	0.004203509	KH domain-containing protein
Glyma.13G147100	Chr13:26033663-26033882	IntronR	0.004324314	Acyl-CoA N-acyltransferase
Glyma.08G200500	Chr08:16221724-16221810	IntronR	0.004397768	poly(A) polymerase 2
Glyma.14G142500	Chr14:28726364-28726527	IntronR	0.004803411	Sulfite exporter TauE/SafE family protein
Glyma.08G102600	Chr08:7878747-7879122	IntronR	0.004904398	Protein kinase superfamily protein
Glyma.17G259400	Chr17:41356939-41357018	IntronR	0.004920174	RING/FYVE/PHD zinc finger superfamily protein
Glyma.11G002600	Chr11:137785-138366	IntronR	0.004976194	target of rapamycin
Glyma.01G065800	Chr01:10139564-10139943	IntronR	0.005211526	NB-ARC domain-containing resistance protein
Glyma.08G213400	Chr08:17225725-17226069	IntronR	0.005558836	Molecular chaperone Hsp40/DnaJ family protein
Glyma.12G056500	Chr12:4114793-4116205	IntronR	0.00568271	A20/AN1-like zinc finger family protein
Glyma.13G358400	Chr13:44613878-44614393	IntronR	0.005717961	no annotation
Glyma.06G159300	Chr06:13131603-13132003	IntronR	0.006022532	Protein of unknown function (DUF668)
Glyma.15G028500	Chr15:2279503-2279636	IntronR	0.006041528	RNA-binding ASCH domain protein
Glyma.16G136400	Chr16:29351927-29352790	IntronR	0.006051405	anoctamin-like protein
Glyma.11G247900	Chr11:34054176-34054278	IntronR	0.006325855	DNA polymerase alpha 2
Glyma.14G162400	Chr14:39326028-39326234	IntronR	0.006408767	alpha/beta-Hydrolases superfamily protein
Glyma.02G150800	Chr02:15528374-15528509	IntronR	0.00644879	histidine-containing phosphotransfer factor 5
Glyma.14G155700	Chr14:33847823-33848306	IntronR	0.006580586	GDSL-like Lipase/Acylhydrolase protein
Glyma.05G062900	Chr05:5982920-5983116	IntronR	0.006755113	Phosphotyrosine protein phosphatases protein
Glyma.08G102600	Chr08:7877073-7877143	IntronR	0.006881935	Protein kinase superfamily protein
Glyma.17G259400	Chr17:41357130-41357425	IntronR	0.007112451	RING/FYVE/PHD zinc finger superfamily protein
Glyma.03G240100	Chr03:43892443-43892732	IntronR	0.007182209	D-aminoacid aminotransferase-like PLP-dependent enzymes
Glyma.20G158600	Chr20:39715282-39715386	IntronR	0.007261751	Pentatricopeptide repeat superfamily protein
Glyma.09G203500	Chr09:42793129-42793369	IntronR	0	phosphoenolpyruvate carboxykinase 1
Glyma.06G122700	Chr06:10002897-10003157	IntronR	0.007602151	TRICHOME BIREFRINGENCE-LIKE 7
Glyma.09G119200	Chr09:28555207-28555416	IntronR	0.007714305	glycerol-3-phosphate acyltransferase 9
Glyma.16G136500	Chr16:29358468-29358575	IntronR	0.007771416	Exo-1,3-beta-glucosidase
Glyma.05G200800	Chr05:38464206-38464279	IntronR	0.007836127	auxin response factor 2
Glyma.02G225000	Chr02:41247186-41247695	IntronR	0.008090868	Glycosyl hydrolase family protein
Glyma.10G223300	Chr10:45435858-45436172	IntronR	0.008155459	polypyrimidine tract-binding protein 3
Glyma.03G221200	Chr03:42435381-42435686	IntronR	0.008173166	RNA helicase family protein
Glyma.06G070400	Chr06:5396315-5396556	IntronR	0.008343043	Plant Tudor-like RNA-binding protein
Glyma.04G068900	Chr04:5759958-5760168	IntronR	0.008489665	no annotation
Glyma.15G058900	Chr15:4547758-4549085	IntronR	0.008604309	aldehyde dehydrogenase 6B2
Glyma.08G213400	Chr08:17227783-17228235	IntronR	0.008781439	Molecular chaperone Hsp40/DnaJ family protein
Glyma.17G249800	Chr17:40484888-40485619	IntronR	0.008810253	Protein of unknown function (DUF1195)
Glyma.06G070400	Chr06:5395649-5396260	IntronR	0.008904619	Plant Tudor-like RNA-binding protein
Glyma.05G013400	Chr05:1266451-1266528	IntronR	0.008960635	TBP-associated factor 2

Glyma.02G144300	Chr02:14886325-14887506	IntronR	0.008971028	2-oxoglutarate and Fe(II)-dependent oxygenase protein
Glyma.02G153700	Chr02:15779055-15779346	IntronR	0.009111538	Zinc finger C-x8-C-x5-C-x3-H type family protein
Glyma.04G074300	Chr04:6185142-6185387	IntronR	0.009358727	ubiquitin-specific protease 26
Glyma.08G043000	Chr08:3402228-3402314	IntronR	0.009366968	jasmonate-zim-domain protein 3
Glyma.13G357800	Chr13:44553579-44553671	IntronR	0.009624572	Sulfite exporter TauE/SafE family protein
Glyma.01G077400	Chr01:18998404-18998722	IntronR	0.009736047	sulfuric ester hydrolases
Glyma.18G115300	Chr18:14042749-14042886	IntronR	0.010120183	Reticulon family protein
Glyma.15G055000	Chr15:4324769-4324894	IntronR	0.010146052	minichromosome maintenance 9
Glyma.11G222100	Chr11:31734400-31734637	IntronR	0.010278045	Tetratricopeptide repeat (TPR)-like superfamily protein
Glyma.16G127800	Chr16:27985512-27986047	IntronR	0.010394884	fanconi-associated nuclease-like protein
Glyma.20G116400	Chr20:35857046-35857161	IntronR	0.010708435	alpha/beta-Hydrolases superfamily protein
Glyma.06G198600	Chr06:18059552-18059635	IntronR	0.010750634	Ankyrin repeat family protein
Glyma.08G157800	Chr08:12229534-12229622	IntronR	0.011011996	nitrate reductase and xanthine dehydrogenase 2
Glyma.04G067600	Chr04:5645652-5646274	IntronR	0.011116001	DEAD/DEAH box RNA helicase family protein
Glyma.15G246500	Chr15:46957387-46957843	IntronR	0.011269257	embryo defective 3012
Glyma.07G026800	Chr07:2102535-2102616	IntronR	0.0112801	cation exchanger 5
Glyma.13G118800	Chr13:23156482-23156567	IntronR	0.011310005	P-glycoprotein 11
Glyma.06G170700	Chr06:14291421-14292009	IntronR	0.011338659	SNF1-related protein kinase
Glyma.15G055000	Chr15:4323072-4323148	IntronR	0.011516234	minichromosome maintenance 9
Glyma.13G204400	Chr13:31853959-31855142	IntronR	0.011580074	basic helix-loop-helix DNA-binding protein
Glyma.01G238400	Chr01:56285584-56286215	IntronR	0.011638069	Survival SurE-like phosphatase/nucleotidase
Glyma.05G015200	Chr05:1396413-1396492	IntronR	0.011719416	SWAP domain-containing protein
Glyma.04G166300	Chr04:41753852-41754922	IntronR	0.011918597	CCT motif -containing response regulator protein
Glyma.06G018800	Chr06:1425708-1426497	IntronR	0.011919808	Transducin/WD40 repeat-like superfamily protein
Glyma.07G122300	Chr07:14317014-14318371	IntronR	0.011922715	tRNA (guanine-N-7) methyltransferase
Glyma.15G272000	Chr15:50930249-50931772	IntronR	0.011934485	SWAPdomain-containing protein
Glyma.17G173200	Chr17:17885373-17886144	IntronR	0.01197933	dihydroflavonol 4-reductase
Glyma.11G172200	Chr11:18703019-18703098	IntronR	0.012069965	fructose-2,6-bisphosphatase
Glyma.05G013400	Chr05:1268743-1269678	IntronR	0.012149523	TBP-associated factor 2
Glyma.16G127800	Chr16:27984661-27986047	IntronR	0.012207364	fanconi-associated nuclease-like protein
Glyma.14G161600	Chr14:38282593-38283217	IntronR	0.012323571	cyclic nucleotide gated channel 1
Glyma.11G112900	Chr11:8636724-8636845	IntronR	0.012440916	hypothetical protein
Glyma.17G059400	Chr17:4501555-4501653	IntronR	0.012558799	pathogenesis related homeodomain protein A
Glyma.06G070400	Chr06:5395299-5395519	IntronR	0.012896746	Plant Tudor-like RNA-binding protein
Glyma.14G127800	Chr14:20501923-20502262	IntronR	0.013116125	pleiotropic drug resistance 12
Glyma.06G170000	Chr06:14195975-14196224	IntronR	0.013216596	coiled coil protein
Glyma.08G282400	Chr08:38722336-38722488	IntronR	0.013261956	nucleotide-diphospho-sugar transferase family protein
Glyma.12G080900	Chr12:6385715-6385790	IntronR	0.013319154	DNA ligase 1
Glyma.10G163700	Chr10:39760412-39760729	IntronR	0.013491773	phosphoribosylaminoimidazole carboxylase
Glyma.13G343500	Chr13:43490358-43490742	IntronR	0.013768874	S-adenosyl-L-methionine-dependent methyltransferases
Glyma.13G343500	Chr13:43492609-43493229	IntronR	0.014043185	S-adenosyl-L-methionine-dependent methyltransferases
Glyma.07G239600	Chr07:42044196-42044565	IntronR	0.014153103	PAS domain-containing protein

Glyma.05G076800	Chr05:9921648-9921739	IntronR	0.014182259	plastid transcriptionally active 9
Glyma.17G128600	Chr17:10290630-10290937	IntronR	0.014337493	arginosuccinate synthase family
Glyma.08G125500	Chr08:9655869-9655955	IntronR	0.014379911	calpain-type cysteine protease family
Glyma.03G070400	Chr03:16490378-16490935	IntronR	0.014575145	Fes1A
Glyma.03G256800	Chr03:45159583-45160017	IntronR	0.014645138	Glycosyl hydrolase superfamily protein
Glyma.09G055600	Chr09:4965855-4966064	IntronR	0.015228248	heavy metal atpase 2
Glyma.09G244700	Chr09:46699969-46700134	IntronR	0.015528177	Small MutS Related domain-containing protein
Glyma.03G221200	Chr03:42435381-42435680	IntronR	0.015742091	RNA helicase family protein
Glyma.01G238400	Chr01:56285203-56285429	IntronR	0.015885481	Survival protein SurE-like phosphatase/nucleotidase
Glyma.12G084200	Chr12:6735256-6735349	IntronR	0.016073556	alpha-adaptin
Glyma.18G288300	Chr18:56805090-56806648	IntronR	0.016159292	zinc finger C3H1 domain protein
Glyma.15G272000	Chr15:50928462-50928718	IntronR	0.016164427	SWAP domain-containing protein
Glyma.19G161600	Chr19:42232786-42233549	IntronR	0.016245497	magnesium transporter 7
Glyma.05G130300	Chr05:32322456-32322920	IntronR	0.016491048	DNA-directed RNA polymerase II
Glyma.13G340900	Chr13:43283133-43283580	IntronR	0.016911642	ZIP metal ion transporter family
Glyma.09G120600	Chr09:29036000-29036108	IntronR	0.016913453	ENTH/ANTH/VHS superfamily protein
Glyma.03G099400	Chr03:28751558-28751872	IntronR	0.017234544	Ribosomal L18p/L5e family protein
Glyma.20G162400	Chr20:40017718-40017805	IntronR	0.017280228	FAD-binding protein
Glyma.16G014200	Chr16:1243891-1244210	IntronR	0.017325686	exocyst complex component sec10
Glyma.07G058200	Chr07:5199449-5200164	IntronR	0.017794405	SPA (suppressor of phyA-105) protein family
Glyma.14G155900	Chr14:33907604-33909217	IntronR	0.018154166	Zinc finger C-x8-C-x5-C-x3-H type family protein
Glyma.08G225400	Chr08:18256524-18257836	IntronR	0.018163389	Tetratricopeptide repeat (TPR)-like superfamily protein
Glyma.17G086900	Chr17:6713938-6714025	IntronR	0.018301811	kinetochore protein
Glyma.12G088700	Chr12:7274999-7275103	IntronR	0.018442975	bZIP transcription factor family protein
Glyma.07G026800	Chr07:2103383-2103466	IntronR	0.018889539	cation exchanger 5
Glyma.05G217800	Chr05:39787459-39787569	IntronR	0.018903106	Zinc finger, C3HC4 type protein
Glyma.05G201500	Chr05:38515071-38515223	IntronR	0.019528775	Polynucleotide adenylyltransferase family protein
Glyma.10G293900	Chr10:51124620-51124884	IntronR	0.019629773	DnaJ/Hsp40 cysteine-rich domain protein
Glyma.17G038800	Chr17:2875250-2875678	IntronR	0.019657132	Calmodulin-binding transcription activator protein
Glyma.09G006600	Chr09:483935-484542	IntronR	0.019757489	6-N-acetylglucosaminyltransferase family protein
Glyma.17G173200	Chr17:17886265-17887677	IntronR	0.020081678	dihydroflavonol 4-reductase
Glyma.16G136400	Chr16:29352599-29352790	IntronR	0.020163662	anoctamin-like protein
Glyma.13G355900	Chr13:44437029-44437574	IntronR	0.020472312	Predicted eukaryotic LigT
Glyma.19G161600	Chr19:42232245-42232390	IntronR	0.020599042	magnesium transporter 7
Glyma.15G058900	Chr15:4546938-4547146	IntronR	0.021150441	aldehyde dehydrogenase 6B2
Glyma.10G035200	Chr10:3072182-3072331	IntronR	0.021897046	alpha/beta-Hydrolases superfamily protein
Glyma.17G059400	Chr17:4501555-4501777	IntronR	0.021902393	pathogenesis related homeodomain protein A
Glyma.15G030400	Chr15:2442620-2442723	IntronR	0.022249878	ARM repeat superfamily protein
Glyma.05G188500	Chr05:37447129-37447555	IntronR	0.022300488	carbamoyl phosphate synthetase A
Glyma.05G021700	Chr05:1909685-1909767	IntronR	0.02237295	Protein of unknown function, transmembrane-40
Glyma.09G192500	Chr09:41712754-41712913	IntronR	0.022465392	Major facilitator superfamily protein
Glyma.03G146200	Chr03:36154806-36155107	IntronR	0.022517919	spindle pole body component 98

Glyma.02G078500	Chr02:6769545-6770261	IntronR	0.022943292	Protein kinase superfamily protein
Glyma.13G091700	Chr13:20654844-20654949	IntronR	0.023217447	Alg9-like mannosyltransferase family
Glyma.08G270900	Chr08:35282922-35282991	IntronR	0.023582071	transferases, transferring acyl groups
Glyma.07G026800	Chr07:2103383-2103470	IntronR	0.023859573	cation exchanger 5
Glyma.15G051700	Chr15:4075721-4076064	IntronR	0.023947593	Transducin/WD40 repeat-like superfamily protein
Glyma.11G038800	Chr11:2769009-2769099	IntronR	0.023956398	Protein kinase superfamily protein
Glyma.14G212900	Chr14:47807598-47808187	IntronR	0.02398043	zinc ion binding
Glyma.15G236300	Chr15:44492231-44494053	IntronR	0.024118949	Tetratricopeptide repeat like superfamily protein
Glyma.06G095500	Chr06:7542698-7543411	IntronR	0.024274768	pyridoxin (pyrodoxamine) 5\'-phosphate oxidase
Glyma.17G249800	Chr17:40484976-40485619	IntronR	0.024714757	Protein of unknown function (DUF1195)
Glyma.13G150200	Chr13:26366845-26366945	IntronR	0.024979596	KDO transferase A
Glyma.20G248000	Chr20:47740448-47740715	IntronR	0.025057613	lipase class 3 family protein
Glyma.08G318000	Chr08:43742047-43743320	IntronR	0.025588651	NB-ARC domain-containing protein
Glyma.08G318000	Chr08:43739243-43739845	IntronR	0.02584407	NB-ARC domain-containing protein
Glyma.16G094900	Chr16:16726476-16726545	IntronR	0.025925084	x-ray induced transcript 1
Glyma.08G040200	Chr08:3166738-3166897	IntronR	0.026001651	DNAse I-like superfamily protein
Glyma.19G009400	Chr19:892299-892621	IntronR	0.026144934	HD domain-containing metal-dependent protein
Glyma.14G078300	Chr14:6627048-6627173	IntronR	0.026199859	no annotation
Glyma.01G158400	Chr01:49636600-49637406	IntronR	0.026494859	MUTL protein homolog 3
Glyma.08G111200	Chr08:8560838-8561045	IntronR	0.026710958	RAB geranylgeranyl transferase beta subunit 1
Glyma.08G305500	Chr08:42366830-42367647	IntronR	0.026717991	fimbrin-like protein 2
Glyma.16G127800	Chr16:27984661-27985211	IntronR	0.026875661	fanconi-associated nuclease-like protein
Glyma.09G210300	Chr09:43445246-43445844	IntronR	0.02730785	protein dimerizations
Glyma.13G150200	Chr13:26367009-26367092	IntronR	0.027641057	KDO transferase A
Glyma.05G209900	Chr05:39184773-39185503	IntronR	0.027747767	PLAC8 family protein
Glyma.15G076300	Chr15:5879238-5879311	IntronR	0.028853528	protease-related
Glyma.19G208300	Chr19:46328391-46328482	IntronR	0.028906017	peroxin 6
Glyma.04G215300	Chr04:48685951-48686038	IntronR	0.029795695	Protein of unknown function (DUF167)
Glyma.06G095500	Chr06:7545743-7545907	IntronR	0.030003923	pyridoxin (pyrodoxamine) 5\'-phosphate oxidase
Glyma.10G257100	Chr10:48362131-48363272	IntronR	0.030715219	cyclin-related
Glyma.03G250600	Chr03:44634522-44634793	IntronR	0.03181868	myb domain protein 15
Glyma.18G212300	Chr18:49880928-49881278	IntronR	0.031837292	TATA binding protein 2
Glyma.04G098900	Chr04:8995561-8995639	IntronR	0.031994429	isoamylase 3
Glyma.07G270100	Chr07:44283511-44283587	IntronR	0.032239664	snRNA activating complex family protein
Glyma.04G098900	Chr04:9000980-9001115	IntronR	0.032440468	isoamylase 3
Glyma.05G150200	Chr05:34409265-34409871	IntronR	0.032632997	Pentatricopeptide repeat superfamily protein
Glyma.06G095500	Chr06:7542231-7542442	IntronR	0.032661941	pyridoxin (pyrodoxamine) 5\'-phosphate oxidase
Glyma.20G170000	Chr20:40756280-40756548	IntronR	0.032772653	gigantea protein (GI)
Glyma.19G056700	Chr19:10305680-10305778	IntronR	0.032833331	golgin candidate 6
Glyma.03G220900	Chr03:42409259-42409342	IntronR	0.033049098	RNA binding;abscisic acid binding
Glyma.14G223500	Chr14:48879659-48880050	IntronR	0.033297124	catalase 2
Glyma.02G093000	Chr02:8255129-8255318	IntronR	0.033347958	Prenyltransferase family protein

Glyma.20G094800	Chr20:33790799-33790873	IntronR	0.033614604	Domain of unknown function (DUF1995)
Glyma.01G073000	Chr01:13463156-13463229	IntronR	0.033886776	FAD-linked oxidases family protein
Glyma.05G197300	Chr05:38158745-38159148	IntronR	0.034048759	DDT domain-containing protein
Glyma.02G296300	Chr02:47400196-47400605	IntronR	0.034261881	fimbrin 1
Glyma.18G206500	Chr18:49028233-49028341	IntronR	0.034784093	MATE efflux family protein
Glyma.05G076800	Chr05:9921648-9921737	IntronR	0.035379042	plastid transcriptionally active 9
Glyma.07G271000	Chr07:44349717-44349972	IntronR	0.035749009	yacP-like NYN domain protein
Glyma.18G297800	Chr18:57504266-57504825	IntronR	0.03624589	Flavodoxin family protein
Glyma.08G109600	Chr08:8410047-8410281	IntronR	0.036994953	RNA recognition motif (RRM)-containing protein
Glyma.15G047400	Chr15:3756172-3756513	IntronR	0.037247456	RHOMBOID-like protein 10
Glyma.18G261100	Chr18:54705796-54705884	IntronR	0.037555461	RNA-binding KH domain-containing protein
Glyma.03G076300	Chr03:18839602-18839963	IntronR	0.03759306	Thioredoxin superfamily protein
Glyma.18G206500	Chr18:49026479-49027133	IntronR	0.037686298	MATE efflux family protein
Glyma.12G053000	Chr12:3813644-3814066	IntronR	0.038107668	hypothetical protein
Glyma.10G163700	Chr10:39763573-39763928	IntronR	0.038405508	phosphoribosylaminoimidazole carboxylase
Glyma.15G166700	Chr15:14534749-14535284	IntronR	0.039273344	DNA ligase IV
Glyma.06G265100	Chr06:45240868-45240944	IntronR	0.039483368	Glyma.06G265100.1.p
Glyma.20G170000	Chr20:40771817-40771999	IntronR	0.039538313	gigantea protein (GI)
Glyma.05G045000	Chr05:4014637-4015221	IntronR	0.039986543	Protein kinase superfamily protein
Glyma.10G218500	Chr10:45050929-45051404	IntronR	0.040119887	transducin family protein / WD-40 repeat family protein
Glyma.07G026800	Chr07:2100758-2102419	IntronR	0.040367622	cation exchanger 5
Glyma.07G234200	Chr07:41527774-41527878	IntronR	0.041384459	squamosa promoter binding protein-like 1
Glyma.10G268700	Chr10:49126069-49126169	IntronR	0.042408546	oxygenase superfamily protein
Glyma.20G023100	Chr20:2447937-2448381	IntronR	0.043053177	Pentatricopeptide repeat (PPR) superfamily protein
Glyma.08G245100	Chr08:21174019-21174120	IntronR	0.043247238	RS2-interacting KH protein
Glyma.16G136400	Chr16:29351927-29353106	IntronR	0.043316488	anoctamin-like protein
Glyma.13G193700	Chr13:30687009-30687280	IntronR	0.043727959	TGACG motif-binding factor 6
Glyma.15G047400	Chr15:3756926-3757415	IntronR	0.044490571	RHOMBOID-like protein 10
Glyma.15G099500	Chr15:7750574-7750660	IntronR	0.044494329	Major facilitator superfamily protein
Glyma.13G355900	Chr13:44436488-44436915	IntronR	0.045982743	Predicted eukaryotic LigT
Glyma.10G221400	Chr10:45278659-45278750	IntronR	0.046146225	carboxypeptidase D, putative
Glyma.19G208300	Chr19:46323750-46323835	IntronR	0.046259863	peroxin 6
Glyma.20G210700	Chr20:44717585-44717674	IntronR	0.046272727	Protein of unknown function (DUF1012)
Glyma.13G187200	Chr13:30107690-30108645	IntronR	0.046705712	Proteasome component (PCI) domain protein
Glyma.12G159000	Chr12:27383218-27383323	IntronR	0.047425139	O-fucosyltransferase family protein
Glyma.17G033400	Chr17:2446534-2446951	IntronR	0.047457091	high chlorophyll fluorescent 107
Glyma.19G233500	Chr19:48327449-48328669	IntronR	0.048376888	T-box protein
Glyma.04G255700	Chr04:52209595-52209690	ExonS	1.67155E-06	U2 snRNP auxilliary factor
Glyma.04G255700	Chr04:52209595-52209693	ExonS	1.93611E-05	U2 snRNP auxilliary factor
Glyma.04G255700	Chr04:52209552-52209693	ExonS	2.50927E-05	U2 snRNP auxilliary factor
Glyma.04G229300	Chr04:49819765-49820029	ExonS	3.42754E-05	SER/ARG-rich protein 34A
Glyma.05G137400	Chr05:33001010-33001076	ExonS	7.97089E-05	ZIP metal ion transporter family

Glyma.01G168300	Chr01:50602556-50602634	ExonS	7.97512E-05	no annotation
Glyma.03G167000	Chr03:38143924-38144009	ExonS	0.000178384	Tetratricopeptide repeat (TPR)-like superfamily protein
Glyma.09G116600	Chr09:27017507-27017565	ExonS	0.000350498	Chaperone DnaJ-domain superfamily protein
Glyma.04G229300	Chr04:49819958-49820029	ExonS	0.000379288	SER/ARG-rich protein 34A
Glyma.18G249600	Chr18:53606034-53606084	ExonS	0.000400415	ELMO/CED-12 family protein
Glyma.03G205500	Chr03:41351586-41351789	ExonS	0.000400525	FAD-linked oxidases family protein
Glyma.05G230000	Chr05:40800891-40801009	ExonS	0.002192131	nuclear fusion defective related protein
Glyma.19G110500	Chr19:36435084-36435180	ExonS	0.002258451	PHD finger protein-related
Glyma.14G073100	Chr14:6141095-6141151	ExonS	0.002627868	pfkB-like carbohydrate kinase family protein
Glyma.11G111600	Chr11:8511837-8511945	ExonS	0.002995152	AT3G51100.1
Glyma.20G170000	Chr20:40760343-40760561	ExonS	0.003510572	gigantea protein (GI)
Glyma.01G168300	Chr01:50602463-50602634	ExonS	0.003537395	no annotation
Glyma.15G236500	Chr15:44561862-44562181	ExonS	0.003699743	Transducin/WD40 repeat-like superfamily protein
Glyma.19G247100	Chr19:49359612-49359942	ExonS	0.003767676	Profilin family protein
Glyma.02G140200	Chr02:14536279-14537100	ExonS	0.004764651	RNA-binding KH domain-containing protein
Glyma.13G286300	Chr13:38697625-38697687	ExonS	0.005002648	HSP20-like chaperones superfamily protein
Glyma.19G233100	Chr19:48310608-48310695	ExonS	0.007815293	AT1G76480.2
Glyma.19G108300	Chr19:36082360-36082410	ExonS	0.008093826	Transmembrane and coiled-coil protein
Glyma.06G092100	Chr06:7272138-7272560	ExonS	0.00874713	Phosphoglycerate mutase family protein
Glyma.06G093100	Chr06:7332041-7332139	ExonS	0.011154587	phosphoglucose isomerase 1
Glyma.14G064900	Chr14:5332779-5333094	ExonS	0.011540939	Mitochondrial substrate carrier family protein
Glyma.07G257000	Chr07:43319967-43320086	ExonS	0.011553892	SNARE associated Golgi protein family
Glyma.10G188600	Chr10:42198049-42198101	ExonS	0.01228201	TRAF-like superfamily protein
Glyma.17G150300	Chr17:12463941-12464117	ExonS	0.01240037	nicotinamide mononucleotide adenyltransferase
Glyma.05G021700	Chr05:1908654-1909011	ExonS	0.012567209	Protein of unknown function, transmembrane-40
Glyma.06G092100	Chr06:7272031-7272560	ExonS	0.01308404	Phosphoglycerate mutase family protein
Glyma.20G107300	Chr20:34991700-34991857	ExonS	0.01332468	ubiquitin-conjugating enzyme 23
Glyma.06G092100	Chr06:7272023-7272560	ExonS	0.013342804	Phosphoglycerate mutase family protein
Glyma.07G257000	Chr07:43319967-43320123	ExonS	0.014790607	SNARE associated Golgi protein family
Glyma.20G130500	Chr20:37137514-37137583	ExonS	0.015435538	caspase-6 protein
Glyma.09G257500	Chr09:47651071-47651625	ExonS	0.016545171	jagunal-like protein
Glyma.09G257500	Chr09:47651071-47651617	ExonS	0.017026977	jagunal-like protein
Glyma.02G140200	Chr02:14536279-14536441	ExonS	0.01714215	RNA-binding KH domain-containing protein
Glyma.19G081300	Chr19:29357525-29357609	ExonS	0.017332076	dyggve-melchior-clausen syndrome protein
Glyma.19G108300	Chr19:36082365-36082410	ExonS	0.017489425	Transmembrane and coiled-coil protein
Glyma.10G134600	Chr10:36082928-36083068	ExonS	0.01797917	Ribosomal protein L14p/L23e family protein
Glyma.20G130500	Chr20:37138165-37138182	ExonS	0.019554003	caspase-6 protein
Glyma.09G116600	Chr09:27022756-27022971	ExonS	0.020742096	Chaperone DnaJ-domain superfamily protein
Glyma.17G150300	Chr17:12462526-12462611	ExonS	0.021431013	nicotinamide mononucleotide adenyltransferase
Glyma.03G248200	Chr03:44472464-44472634	ExonS	0.022165198	RNA-binding KH domain-containing protein
Glyma.09G257500	Chr09:47651071-47651597	ExonS	0.022220694	jagunal-like protein
Glyma.11G208400	Chr11:29817167-29817331	ExonS	0.022700405	Cytochrome c oxidase biogenesis protein Cmc1-like

Glyma.10G134600	Chr10:36082921-36083068	ExonS	0.023678044	Ribosomal protein L14p/L23e family protein
Glyma.10G214400	Chr10:44651299-44651458	ExonS	0.023988263	downstream neighbor of Son
Glyma.05G021700	Chr05:1908654-1908711	ExonS	0.024432691	Protein of unknown function, transmembrane-40
Glyma.10G214400	Chr10:44652585-44652717	ExonS	0.025578366	downstream neighbor of Son
Glyma.06G026000	Chr06:2000277-2000889	ExonS	0.026738029	bZIP transcription factor family protein
Glyma.02G035800	Chr02:3306992-3307334	ExonS	0.029491688	SBP (S-ribonuclease binding protein) family protein
Glyma.03G167000	Chr03:38143762-38144009	ExonS	0.030305047	Tetratricopeptide repeat (TPR)-like superfamily protein
Glyma.01G000200	Chr01:64055-64199	ExonS	0.032925257	no annotation
Glyma.03G179400	Chr03:39163917-39164064	ExonS	0.03334337	no annotation
Glyma.13G244500	Chr13:35389359-35389373	ExonS	0.033739313	Protein of unknown function (DUF707)
Glyma.01G006500	Chr01:637998-638071	ExonS	0.03407251	nuclear RNA polymerase C2
Glyma.01G099000	Chr01:33001179-33001248	ExonS	0.034126176	acyl-CoA oxidase 4
Glyma.01G006500	Chr01:638001-638071	ExonS	0.034211965	nuclear RNA polymerase C2
Glyma.03G080300	Chr03:21087151-21087288	ExonS	0.034305433	E3 Ubiquitin ligase family protein
Glyma.17G142500	Chr17:11585924-11585985	ExonS	0.035548382	hypothetical protein
Glyma.01G000200	Chr01:65205-65537	ExonS	0.035778508	no annotation
Glyma.06G324000	Chr06:51173936-51174031	ExonS	0.036315131	U2 snRNP auxilliary factor
Glyma.10G239100	Chr10:46775921-46775998	ExonS	0.037918062	arginine/serine-rich splicing factor 35
Glyma.06G233300	Chr06:37299535-37299658	ExonS	0.039230366	PHD finger family protein
Glyma.20G130500	Chr20:37137514-37137583	ExonS	0.039234407	caspase-6 protein
Glyma.06G198600	Chr06:18059636-18059686	ExonS	0.039839694	Ankyrin repeat family protein
Glyma.08G345300	Chr08:46026550-46026605	ExonS	0.042286505	RAB GTPase homolog A5B
Glyma.06G092100	Chr06:7272490-7272560	ExonS	0.043394811	Phosphoglycerate mutase family protein
Glyma.14G155700	Chr14:33847913-33848626	ExonS	0.044224205	GDSL-like Lipase/Acylhydrolase superfamily protein
Glyma.05G060100	Chr05:5601423-5601463	ExonS	0.044982796	hypothetical protein
Glyma.11G169700	Chr11:18043941-18044007	ExonS	0.046652512	fructose-2,6-bisphosphatase
Glyma.11G169700	Chr11:18043941-18044008	ExonS	0.046662324	fructose-2,6-bisphosphatase
Glyma.17G142500	Chr17:11585576-11585813	ExonS	0.047201732	hypothetical protein
Glyma.03G080300	Chr03:21087151-21087270	ExonS	0.047257763	E3 Ubiquitin ligase family protein
Glyma.06G233300	Chr06:37299535-37299641	ExonS	0.048746155	PHD finger family protein
Glyma.12G088300	Chr12:7199834-7200016	ExonS	0.049446381	NAD+ ADP-ribosyltransferases
Glyma.04G255700	Chr04:52209595-52209979	AltD	7.43098E-07	U2 snRNP auxilliary factor
Glyma.03G205500	Chr03:41351586-41351885	AltD	7.81032E-07	FAD-linked oxidases family protein
Glyma.04G255700	Chr04:52209552-52209979	AltD	9.16382E-07	U2 snRNP auxilliary factor
Glyma.05G044200	Chr05:3957779-3958732	AltD	3.24425E-05	MA3 domain-containing protein
Glyma.06G324000	Chr06:51173647-51174031	AltD	6.00882E-05	U2 snRNP auxilliary factor
Glyma.18G020900	Chr18:1530121-1530366	AltD	0.000130404	bZIP transcription factor family protein
Glyma.02G078500	Chr02:6765903-6765944	AltD	0.00029019	Protein kinase superfamily protein
Glyma.01G219700	Chr01:54909794-54909833	AltD	0.000385979	Polynucleotidyl transferase
Glyma.07G152200	Chr07:18493228-18493240	AltD	0.000663684	electron transport SCO1/SenC family protein
Glyma.17G075200	Chr17:5904575-5904579	AltD	0.001185121	LJRHL1-like 1
Glyma.11G216200	Chr11:31041560-31042169	AltD	0.001937356	ALWAYS EARLY 4

Glyma.16G035400	Chr16:3359009-3359407	AltD	0.001974304	Cyclin/Brf1-like TBP-binding protein
Glyma.05G076800	Chr05:9921738-9921738	AltD	0.002828203	plastid transcriptionally active 9
Glyma.06G072200	Chr06:5539703-5539774	AltD	0.003942369	WD40/YVTN repeat-like-containing domain protein
Glyma.06G128700	Chr06:10585376-10585401	AltD	0.00443325	Calcium-binding EF-hand family protein
Glyma.19G228600	Chr19:48002720-48002738	AltD	0.004833311	Peptide chain release factor 2
Glyma.17G059400	Chr17:4501654-4501776	AltD	0.005558097	pathogenesis related homeodomain protein A
Glyma.18G051700	Chr18:4468940-4469223	AltD	0.006289876	DEA(D/H)-box RNA helicase family protein
Glyma.06G072200	Chr06:5539693-5539774	AltD	0.008592102	WD40/YVTN repeat-like-containing domain protein
Glyma.10G179700	Chr10:41304340-41304357	AltD	0.009926146	Protein of unknown function (DUF1012)
Glyma.19G210000	Chr19:46435449-46435607	AltD	0.010371681	asparagine-linked glycosylation 3
Glyma.13G343500	Chr13:43492609-43492888	AltD	0.010486173	S-adenosyl-L-methionine-dependent methyltransferases
Glyma.16G080900	Chr16:8640359-8641593	AltD	0.010786117	beta-hydroxyisobutyryl-CoA hydrolase 1
Glyma.06G083000	Chr06:6371429-6371972	AltD	0.012239269	alpha/beta-Hydrolases superfamily protein
Glyma.08G305500	Chr08:42366830-42367023	AltD	0.012676051	fimbrin-like protein 2
Glyma.05G103200	Chr05:27360947-27360969	AltD	0.013929841	Subtilase family protein
Glyma.04G122600	Chr04:15770767-15770802	AltD	0.013978683	elongation factor P (EF-P) family protein
Glyma.18G020900	Chr18:1535336-1535386	AltD	0.015229121	bZIP transcription factor family protein
Glyma.09G227900	Chr09:45218137-45218269	AltD	0.017688816	2-oxoglutarate and Fe(II)-dependent oxygenase protein
Glyma.14G064900	Chr14:5332449-5333094	AltD	0.017826477	Mitochondrial substrate carrier family protein
Glyma.15G240800	Chr15:45728538-45728580	AltD	0.01816619	pfkB-like carbohydrate kinase family protein
Glyma.09G244700	Chr09:46700115-46700133	AltD	0.018772216	smr (Small MutS Related) domain-containing protein
Glyma.04G189800	Chr04:46062630-46063191	AltD	0.020293868	Rhodanese/Cell cycle control phosphatase
Glyma.05G076800	Chr05:9922075-9922122	AltD	0.021157714	plastid transcriptionally active 9
Glyma.02G078500	Chr02:6765232-6765661	AltD	0.022357065	Protein kinase superfamily protein
Glyma.11G117600	Chr11:8940927-8941084	AltD	0.02422411	cold, circadian rhythm, and rna binding 2
Glyma.09G227900	Chr09:45225332-45225693	AltD	0.026205469	2-oxoglutarate and Fe(II)-dependent oxygenase protein
Glyma.08G184900	Chr08:14827237-14827242	AltD	0.034594531	Acyl transferase/acyl hydrolase/lysophospholipase
Glyma.18G099900	Chr18:10473860-10473863	AltD	0.034759967	Biotin/lipoate A/B protein ligase family
Glyma.05G060100	Chr05:5603362-5603422	AltD	0.041449537	hypothetical protein
Glyma.18G020900	Chr18:1530367-1530708	AltD	0.04291379	bZIP transcription factor family protein
Glyma.01G186000	Chr01:52130412-52130425	AltD	0.043507849	CLK4-associating serine/arginine-rich protein
Glyma.20G130500	Chr20:37137192-37137233	AltD	0.046548243	caspase-6 protein
Glyma.08G250900	Chr08:21989767-21990802	AltD	0.048372707	Regulator of chromosome condensation protein
Glyma.07G184100	Chr07:35177285-35177289	AltD	0.048509944	heat shock protein
Glyma.05G078300	Chr05:10444106-10444120	AltD	0.048740022	sulfite oxidase
Glyma.07G124200	Chr07:14739412-14739415	AltD	0.049425928	bacterial hemolysin-related
Glyma.16G129000	Chr16:28117730-28117890	AltA	4.2557E-09	zinc finger (Ran-binding) family protein
Glyma.16G129000	Chr16:28117730-28117873	AltA	3.43324E-09	zinc finger (Ran-binding) family protein
Glyma.15G167700	Chr15:14868597-14868615	AltA	3.07906E-05	hypothetical protein
Glyma.18G098400	Chr18:10248356-10248365	AltA	3.76571E-05	alpha/beta-Hydrolases superfamily protein
Glyma.09G281300	Chr09:49660144-49660334	AltA	6.13544E-05	RING/U-box superfamily protein
Glyma.19G151500	Chr19:41190321-41190541	AltA	6.4951E-05	coiled-coil protein

Glyma.13G365200	Chr13:45138028-45138032	AltA	0.000102122	hypothetical protein
Glyma.05G029200	Chr05:2510917-2511234	AltA	0.000158956	aldehyde dehydrogenase 12A1
Glyma.11G038800	Chr11:2769927-2769939	AltA	0.000210747	Protein kinase superfamily protein
Glyma.05G029200	Chr05:2510920-2511234	AltA	0.000281684	aldehyde dehydrogenase 12A1
Glyma.04G237300	Chr04:50589694-50590145	AltA	0.00027784	spermidine synthase 3
Glyma.04G237300	Chr04:50589707-50590145	AltA	0.000378605	spermidine synthase 3
Glyma.14G136700	Chr14:24187419-24187427	AltA	0.000544358	serine/arginine-rich 22
Glyma.05G167600	Chr05:35821562-35821568	AltA	0.000717603	Integrin-linked protein kinase family
Glyma.20G044800	Chr20:8270403-8270417	AltA	0.00076423	Late embryogenesis abundant protein, group 2
Glyma.14G022900	Chr14:1624756-1624785	AltA	0.000853442	RNI-like superfamily protein
Glyma.08G308200	Chr08:42670000-42670013	AltA	0.001179706	glucan synthase-like 7
Glyma.12G038800	Chr12:2807951-2807970	AltA	0.00129355	hypothetical protein
Glyma.04G194900	Chr04:46666324-46666329	AltA	0.001368073	AAA-type ATPase family protein
Glyma.14G075500	Chr14:6327078-6327406	AltA	0.001732	O-Glycosyl hydrolases family 17 protein
Glyma.04G233100	Chr04:50139857-50139894	AltA	0.002223342	nucleotide binding;nucleic acid binding
Glyma.17G234900	Chr17:39009690-39009708	AltA	0.002304171	transmembrane protein
Glyma.19G056700	Chr19:10307133-10307135	AltA	0.002851738	golgin candidate 6
Glyma.08G276400	Chr08:36959649-36960069	AltA	0.003573467	aldehyde dehydrogenase 6B2
Glyma.08G225400	Chr08:18256524-18256528	AltA	0.004460737	Tetratricopeptide repeat (TPR)-like superfamily protein
Glyma.05G212000	Chr05:39347454-39347469	AltA	0.00448554	Transducin/WD40 repeat-like superfamily protein
Glyma.05G029200	Chr05:2510917-2511231	AltA	0.005946411	aldehyde dehydrogenase 12A1
Glyma.10G047800	Chr10:4299366-4300079	AltA	0.006972846	Ypt/Rab-GAP domain of gyp1p superfamily protein
Glyma.10G252200	Chr10:48007864-48007891	AltA	0.006944242	Chaperone DnaJ-domain superfamily protein
Glyma.03G130000	Chr03:34468927-34469322	AltA	0.006849467	response regulator 9
Glyma.08G067100	Chr08:5159571-5159578	AltA	0.007353615	dynamin-related protein 3A
Glyma.13G348300	Chr13:43843216-43843223	AltA	0.007761809	serine/threonine phosphatase 7
Glyma.15G208200	Chr15:29881988-29882013	AltA	0.008128978	FAD-dependent oxidoreductase family protein
Glyma.05G029200	Chr05:2510920-2511231	AltA	0.010405182	aldehyde dehydrogenase 12A1
Glyma.20G064600	Chr20:22388067-22388074	AltA	0.010792407	RING/U-box superfamily protein
Glyma.19G233500	Chr19:48327133-48328035	AltA	0.012361844	T-box protein
Glyma.06G129100	Chr06:10614137-10614144	AltA	0.012899265	CLP protease regulatory subunit X
Glyma.10G047800	Chr10:4299366-4300095	AltA	0.013848209	Ypt/Rab-GAP domain of gyp1p superfamily protein
Glyma.16G179800	Chr16:34025424-34025433	AltA	0.014685614	Class II aaRS and biotin synthetases superfamily protein
Glyma.12G242500	Chr12:40081415-40081428	AltA	0.01437666	S-adenosyl-L-methionine-dependent methyltransferases
Glyma.02G038600	Chr02:3595987-3596004	AltA	0.014590197	MATE efflux family protein
Glyma.08G073500	Chr08:5615475-5615480	AltA	0.015374196	no annotation
Glyma.04G029000	Chr04:2355194-2355454	AltA	0.015970785	Tetrapyrrole (Corrin/Porphyrin) Methylases
Glyma.20G198100	Chr20:43550688-43550721	AltA	0.016745445	plastid transcriptionally active 5
Glyma.19G233500	Chr19:48327133-48328331	AltA	0.017139303	T-box protein
Glyma.13G150200	Chr13:26367831-26367835	AltA	0.017500251	KDO transferase A
Glyma.11G038800	Chr11:2769927-2770583	AltA	0.018627277	Protein kinase superfamily protein
Glyma.08G077100	Chr08:5859054-5859084	AltA	0.018870344	Putative serine esterase family protein

Glyma.08G283400	Chr08:38971998-38972172	AltA	0.019201835	Clathrin adaptor complexes medium subunit protein
Glyma.06G135500	Chr06:11134079-11134088	AltA	0.019706349	SER/ARG-rich protein 34A
Glyma.10G009700	Chr10:914910-915259	AltA	0.020192273	Polyketide cyclase/dehydrase and lipid transport protein
Glyma.19G233500	Chr19:48327449-48328035	AltA	0.022657217	T-box protein
Glyma.11G110800	Chr11:8459528-8459542	AltA	0.023215588	ATP binding microtubule motor family protein
Glyma.20G198100	Chr20:43550652-43550687	AltA	0.024577216	plastid transcriptionally active 5
Glyma.04G237300	Chr04:50590114-50590145	AltA	0.026286821	spermidine synthase 3
Glyma.05G245000	Chr05:41912834-41912875	AltA	0.027318348	Protein of unknown function (DUF1295)
Glyma.08G211300	Chr08:17061605-17062025	AltA	0.027548287	O-fucosyltransferase family protein
Glyma.08G022600	Chr08:1819953-1820522	AltA	0.027822623	Pleckstrin homology (PH) domain-containing protein
Glyma.13G058600	Chr13:15602573-15602958	AltA	0.029783332	casein lytic proteinase B3
Glyma.16G129000	Chr16:28117702-28117873	AltA	0.030179306	zinc finger (Ran-binding) family protein
Glyma.16G129000	Chr16:28117702-28117890	AltA	0.031050156	zinc finger (Ran-binding) family protein
Glyma.19G233500	Chr19:48327133-48327463	AltA	0.031998054	T-box protein
Glyma.19G233500	Chr19:48327449-48327463	AltA	0.033793038	T-box protein
Glyma.11G038800	Chr11:2769940-2770583	AltA	0.03463713	Protein kinase superfamily protein
Glyma.04G237300	Chr04:50589694-50590113	AltA	0.035117451	spermidine synthase 3
Glyma.04G112600	Chr04:12612004-12612819	AltA	0.036694488	actin-related protein 8
Glyma.19G233500	Chr19:48327133-48327473	AltA	0.037045519	T-box protein
Glyma.16G164900	Chr16:32412603-32412604	AltA	0.038831453	NagB/RpiA/CoA transferase-like superfamily protein
Glyma.19G233500	Chr19:48327133-48327448	AltA	0.039761628	T-box protein
Glyma.08G022600	Chr08:1819710-1819714	AltA	0.041539006	Pleckstrin homology (PH) domain-containing protein
Glyma.19G233500	Chr19:48327449-48328331	AltA	0.042856831	T-box protein
Glyma.16G136400	Chr16:29351927-29352904	AltA	0.04493348	anoctamin-like protein
Glyma.16G136400	Chr16:29351927-29352595	AltA	0.047956884	anoctamin-like protein
Glyma.13G150200	Chr13:26369281-26369316	AltA	0.048102613	KDO transferase A
Glyma.11G236300	Chr11:33115165-33115170	AltA	0.002930043	bZIP transcription factor family protein
Glyma.01G097200	Chr01:32006778-32006844	AltA	0.014420258	SPT2 chromatin protein
Glyma.19G006000	Chr19:558635-558672	AltA	0.015531721	DEAD box RNA helicase family protein
Glyma.19G000200	Chr19:19425-19473	AltA	0.019438547	ferredoxin/thioredoxin reductase subunit A
Glyma.13G223900	Chr13:33674003-33674537	AltA	0.024050983	MAC/Perforin domain-containing protein
Glyma.01G223900	Chr01:55261523-55261542	AltA	0.026895785	Galactose mutarotase-like superfamily protein
Glyma.13G202400	Chr13:31610004-31610510	AltA	0.034452114	folate-sensitive fragile site protein
Glyma.09G136800	Chr09:33866652-33866662	AltA	0.048553	GPI-anchored adhesin-like protein
Glyma.09G051200	Chr09:4465999-4466039	AltA	0.049687753	phosphorylcholine cytidylyltransferase
Glyma.08G152600	Chr08:11742389-11742504	AltA	1.31829E-05	SWAP domain-containing protein
Glyma.01G240700	Chr01:56446459-56446474	AltA	5.99587E-05	Trimeric LpxA-like enzymes superfamily protein
Glyma.08G111200	Chr08:8561046-8561607	AltA	0.000401521	RAB geranylgeranyl transferase beta subunit 1
Glyma.12G192900	Chr12:35442145-35442311	AltA	0.000504401	no annotation
Glyma.17G233900	Chr17:38946231-38946246	AltA	0.001176959	Transducin family protein / WD-40 repeat family protein
Glyma.09G080100	Chr09:9211397-9211705	AltA	0.001436547	Cytidine/deoxycytidylate deaminase family protein
Glyma.07G166300	Chr07:25959557-25959572	AltA	0.002369194	ARM repeat superfamily protein

Glyma.19G084600	Chr19:30381197-30381199	AltA	0.002496842	global transcription factor group E8
Glyma.04G217100	Chr04:48813725-48813727	AltA	0.006098186	Alba DNA/RNA-binding protein
Glyma.03G099900	Chr03:28892938-28892951	AltA	0.007254352	Protein of unknown function (DUF1005)
Glyma.08G127400	Chr08:9820573-9821411	AltA	0.009798425	Protein kinase superfamily protein
Glyma.04G165600	Chr04:41485330-41485646	AltA	0.013400343	Protein kinase superfamily protein
Glyma.11G104500	Chr11:7930167-7930318	AltA	0.013723341	ARF-GAP domain 2
Glyma.02G149200	Chr02:15346554-15346559	AltA	0.014163524	Tudor/PWWP/MBT domain-containing protein
Glyma.02G308000	Chr02:48202278-48202281	AltA	0.020305555	Ypt/Rab-GAP domain of gyp1p superfamily protein
Glyma.15G076300	Chr15:5879575-5880188	AltA	0.021590807	protease-related
Glyma.10G102900	Chr10:21512329-21513316	AltA	0.022839159	Arginyl-tRNA synthetase, class Ic
Glyma.03G041800	Chr03:5307895-5307911	AltA	0.027453187	hypothetical protein
Glyma.03G041800	Chr03:5307891-5307894	AltA	0.027425627	hypothetical protein
Glyma.01G036200	Chr01:3807676-3807766	AltA	0.032527446	RNI-like superfamily protein
Glyma.03G031700	Chr03:3569688-3569692	AltA	0.032899829	Protein kinase superfamily protein
Glyma.02G225000	Chr02:41246656-41246673	AltA	0.042892923	Glycosyl hydrolase family protein
Glyma.13G314800	Chr13:41007132-41007143	AltA	0.046978892	Leucine-rich repeat (LRR) family protein
Glyma.08G136700	Chr08:10457818-10457831	AltA	0.000434467	Major facilitator superfamily protein
Glyma.05G041800	Chr05:3746880-3746939	AltA	0.000957238	drought-induced 19
Glyma.13G272800	Chr13:37471971-37471978	AltA	0.001429073	ribonuclease H-like superfamily protein
Glyma.07G270300	Chr07:44302165-44302167	AltA	0.001523145	WD40 repeat-like superfamily protein
Glyma.15G135900	Chr15:10963349-10963807	AltA	0.002491315	hydroxyproline-rich glycoprotein family protein
Glyma.05G016400	Chr05:1472246-1472268	AltA	0.021689785	tRNA synthetase beta subunit family protein
Glyma.01G089500	Chr01:27065063-27065069	AltA	0.026928698	SLH domain protein
Glyma.01G089500	Chr01:27065063-27065068	AltA	0.026966726	SLH domain protein
Glyma.01G089500	Chr01:27065063-27065067	AltA	0.027005522	SLH domain protein
Glyma.08G010300	Chr08:811022-811034	AltA	0.036457343	HSP90-like ATPase family protein
Glyma.20G116400	Chr20:35857261-35857513	AltA	0.044071384	alpha/beta-Hydrolases superfamily protein

Supplementary Table 2. A list of changed alternative splicing events

Supplementary Table 3

Gene No.	Protein Sequence
P. sojaeAvr3c	MRVCSVLLVAAAALIATSNAVEPSATSTVEVAEVQARGADKRFLRSLQTEEEQGDS<br>DVNEAEDGSEERGLFAWIKNAVTGDVLLAKANKGDFEMQTKLFKKWIEEKPKVRQ<br>NAIAKIMRDGGRKKYDTVLTAWKYHDKRTANGIGIRGATDDVDELLPGTLIYRAAAG<br>NQGAQSALFSMWIGAEKKTLDTARILLSKSELPAKEYKRLNKAWVQYRRKHK
P. pistaciae	MRVCYALLVAAATLIATGNTVDASATAQVVSPLAVLANAGVRAVDADKRLLRSRQT<br>EEEEEDSDDTEEEDESEERGLNVGVVDDAFANLRSALRSDDDAVAGLLPTSTLTM<br>ANNGNHDMQRQLFLQWLNAKPEVRQKAIATILRNRGDDDFRTLLAAWMHSGERR<br>TRSVGFHGSNYAVDELLPRLLVKKAGAGDPYAQAVLFSKWMAAPSETRDTALKIL<br>HESAQGTRGYETLNAAWISYLRQHLTTYS
P. robiniae	MRVCSVLLVAAAALIAISSAIEPSATSTVKVAEVQARGADKRFLRSHQTEEEQG<br>DSDVNEEEDDSEERGINVDDALAILRGAVKSRNNDAVDALLPHTILAKANNGDFDM<br>QTKLFQQWLKAKPEVRQNAISTIMRNGGYDDYKTLLTAWRYNSERTTSGV<br>GFHGSTKAVDKLLPRGLIDKAALGNVQARETLFGKWIAAPSETRDTALKILHDS<br>GMGKPAYTRLNSAWLRYLNELVK
P. parvispora	MHLPSVLLFAAVLIAHNAAGASTTTQLISSGISPEALQTGIGTRFLRTHPIAEAEP<br>EDRDDAQVKTESEERGIDLKLVDDAVSKIKDAAQNKYAMKVDDLLEPHYLNAAESD<br>KGIQDILFRRWAAAPTEVRKAAISKLTATEDDWTVLLKAWKEYKATKLTAEA<br>VLVPKKADEVLPKSMMLKANDGNHDAQAELFRMWIVAPPRVRQEAIAKVRAD<br>VTYSTLLTAWRFSGARDKAGLDMLGYSIPTLDDLLQKSLLAKAISGDVKNQNVLFS<br>RWAAAHQETRDAALKILRDVGKGTDEYSALNNAWQKYLKILGKTLDD
P. niederhauserii	MRVCSVLLVVAAAVIAISNAAEASTTQLVSPRDVSAIAKVQVVNAAKRFLRSHQ<br>TTEEAGEDTQEEDESEERTLNLNLVDDAVAKFKDVAKHKYDLKVDDLLSPHYLNAA<br>ENDKGIKKILFKRWATAPAEVRESAIKQLAATGEKWSGLLAAWNKYEAKAA<br>TGFPVSASVANKADDLLPKSLVAKANGGDLDMQEKLFKTWIDAAPRIRQDAIE<br>KLKESGNTYSTVLLAWKYSGSRSKAGIDELGFPLRTLDDLLPKGALRKAMDGDVR<br>EQNALFSQWFAAPKETREAALQILFDVGKGTKDYRALNNAWLNYLEKLGR<br>TLD
P. cajani	MRVCSVLLVAAAAVIAVSNAADLSTTQLVYPRGVSAIAEVPVVDAAKRFLRSH<br>QTAEEADEDSDDAQEEDESEERVLNLNLVDDAVAKFKDVAKHKYDLKVDDLLSPY<br>YLNAAESDSGIMKILFQRWSVAPAEVRKTAIQQLAAKGEKWAGLIKAWNQY<br>EAKAATGFPAPAKIAKTADDLLPKTLVAKANGGDLDVQQKLFKTWIDAAPRIR<br>QDAIEKLKEGGNKYRTVLLAWKYSGARYTAGIDELGVPLRTLDDLLPKGALRKAIAG<br>DVREQNALFSQWVAAPRETREAALQILFDVGKGTKDYRTLNNAWRKYLEN<br>LGRTLD
P. vignae	MRVCSVLLVAAAAVIAVSNAADLSTTQLVYPRGVSAIAEVPVVDAAKRFLRSH<br>QTAEEADEDSDDAQEEDESEERALNLNLVDDAVAKFKDVAKHKYDLKVDDLLSPY<br>YLNAAESDSGIMKILFQTWSVAPAEVRKTAIQQLAAKGEKWAGLIKAWNQYE<br>AKAATGFPASAKIAKTADDLLPKTLVEKANGGDLDVQQKLFKTWIDAAPRIRQ<br>DAIEKLKEGGNKYRTVLLAWKYSERGPIEGWHRRAWRPSPDAG
GmSKRP1	MAASSSSLSASDDSSSHRRRRHHRHRRYRDKDSLKIRKKSKSQSQSRGERR<br>RRHHRHSSDSDSYSSSSLSDYSRSESSSDSEHETSHRSKRHKKSDRPKKNKEKD<br>RSKSHRHKRQKHKVKEKQHDERSSSPVQLSKFLGHDKDDGVRRSAVSGKKILLKL<br>EKTKEDKVAESKRNELLNFLNASFD*

GmSKRP2	MAASSSSLSASDDSSSRRRRRHHRHRRGRDKDSLKIRKKSKSQSQSRGKRR<br>RRHYHHSSDSYSSSSLSDYSRSESSSDSEHETSHRSKRHKKSDRPKKNKEKDQS<br>KSHRHKWQKHKVKEKQHDERSSSPVQLSKFLGRDKDDGVRRSAVSGKKILLKLEK<br>TKEDKVAESKRNELLNFLNASFD*
SlSKRP	MAASSSPSASESSSSSSGHRERKRHRRNRKDKDKDSLKIRKKSRSRTKRHRS<br>RHSSSDSYSYSSSSEDYSSSDSEREAVSSSKRHIQKDRGTKKKNKEKGKSHRQK<br>RHKNKIKEKQQVESNGPVQLSKFLGRDKDDGVRRSAVSGKKILLKLDKTK<br>EDKEAESKRNELLKFLNASYD*
AtSKRP	MAASSSSSSSDVSSDSSDSHRRRKDRRHHRRNDRDRDSLKVRKKSRSTS<br>KKRRRRQHSSDSSDSSYSDSSSESSDSEHEKSRRHKKHEKPKKAKDKERSKSHR<br>HKRHKNRERKKGEGEGSSGPVKLSKFLNRDKDDGERRSAVSGKKILLKV<br>DKSKEDKAAESKRNELLKFLNASFD*
NbSKRP	MAASSSSSVSESSSSSSGHGDRKRHRRHCKDKDKDALKVRKKSRSHTKRR<br>RSRHSSCDSYSSSSSSEDYSSSDSEREAVNSSKRHRQKDRATKKKDKERGKSHR<br>QKRHKHKSKEKQQEESSGPVQLSKFLGRDKDDGVRRSAVSGKKILLKLDK<br>TKEDKEAESKRNELLKFLNASYD*
ZmSKRP	MARSPAASSSSYTDSTGSSSDSGSSSSGIDRRRRRHRHRSSRRKEGTSSS<br>ALKARKDRRSRHKRRRHERERRRSASDDDSYSSTSSYDSDREVSGRSRKHKKSS<br>RSRKFRERERSKDRHHRRDKSKHKEKKESEHADGPVQLSKFLGRDKEK<br>EEGTQRSAISGKKIMMKLEKTKEDKAAESKRNELLKFLNASYD*
OsSKRP	MAPPPAAATSSSSSYTDTSGSSSDSSSSSGSDRRRRRARHRSGHRKDAA<br>AAAASSSSALKARKDRRSRHKRRRRERRRSPSDDDSYSSSSSYDSEHEGKSCKH<br>KKSRSSRKSRERERSKDRHSKRDKSKHKEKKDSERTSGPVQLSKFLGRD<br>KDEGVQRSAISGKKIMMKLEKSKEDKQAESKRNELLKFLNASYD*
Gene No.	CDS Sequence
$GmSKRP1^{mut}$	ATGGCTGCTTCTTCATCTTCACTTTCAGCTTCTGATGATTCTTCATCTCATGCTG<br>CTGCTGCTCATCATGCTCATCGCCGATACCGTGATAAGGACTCCCTCAAGATTC<br>GAAAGAAGAGCAAGTCTCAATCACAATCAAGAGGAGAAGCTGCTGCTGCTCAC<br>CACAGGCATTCATCTGATTCAGATTCTTATTCATCTTCATCTCTTTCAGATTACT<br>CAAGATCTGAGTCATCTTCAGATTCTGAACATGAGACTTCACATAGGTCTGCTG<br>CTCATGCTGCTTCAGATGCTCCAAAGGCTAATGCTGAAGCTGATAGATCAGCTT<br>CTCATGCTCATGCTGCTCAAGCTCATGCTGTTGCTGAAGCTCAACATGATGAGA<br>GGTCTTCATCTCCTGTTCAACTTTCTGCTTTTCTTGGAGCTGATGCTGATGATG<br>GTGTTGCTGCTTCAGCTGTTTCTGGTGCTGCTATTCTTTTGGCTCTTGAAGCTA<br>CAGCTGAGGATGCTGTTGCTGAATCAGCTGCTAATGAGCTTTTGAATTTTCTTA<br>ATGCTTCTTTTGAT

Supplementary Table 3. A list of PsAvr3c and GmSKRPs homologous proteins sequence and synthesized $GmSKRP1^{mut}$ CDS sequence.

Supplementary References

1. Jing M*, et al.* A *Phytophthora sojae* effector suppresses endoplasmic reticulum stress-mediated immunity by stabilizing plant Binding immunoglobulin Proteins. *Nature communications* **7**, 11685 (2016).

2. Day IS*, et al.* Interactions of SR45, an SR-like protein, with spliceosomal proteins and an intronic sequence: insights into regulated splicing. *The Plant journal : for cell and molecular biology* **71**, 936-947 (2012).
